# Inhalable Spray-Dried Carriers for Deep Lung Delivery and In Situ Synthesis of Allicin

**DOI:** 10.1021/acsomega.6c04308

**Published:** 2026-08-05

**Authors:** Lucie Mašková, Mária Kalina, Karolína Slonková, Lara Mugrauer, Vojtěch Klimša, Anna Reiserová, Kateřina Slaninová, Denisa Lizoňová, Viola Tokárová, Ondřej Kašpar

**Affiliations:** Department of Chemical Engineering, 534467 University of Chemistry and Technology Prague, Technická 5, Prague 6 166 28, Czech Republic

## Abstract

Allicin exhibits several therapeutic properties, but its clinical use is limited by its high instability and low bioavailability. Furthermore, although pulmonary delivery of allicin offers a promising approach for treating various respiratory diseases, direct exposure of the lungs to allicin through aerosols or vapors carries a significant risk of respiratory tract irritation or tissue damage. Facing these challenges, we propose a novel bioinspired strategy that facilitates the *in situ* synthesis of allicin from its stabilized precursors using the Büchi Mini Spray Dryer equipped with a three-fluid (3F) nozzle to develop inhalable carriers suitable for deep lung delivery. We have identified conditions that ensure successful coencapsulation of both enzyme and substrate within maltodextrin core–shell microparticles, and we have examined the effects of selected additives on allicin yield and aerosolization performance under various storage conditions. Each 3F particle acts as an independent enzyme–substrate system that, upon deposition, dissolves and releases active allicin within the network of the respiratory tract. We believe that such microcarriers may help overcome the challenges of pulmonary delivery of allicin and other unstable compounds.

## Graphical abstract

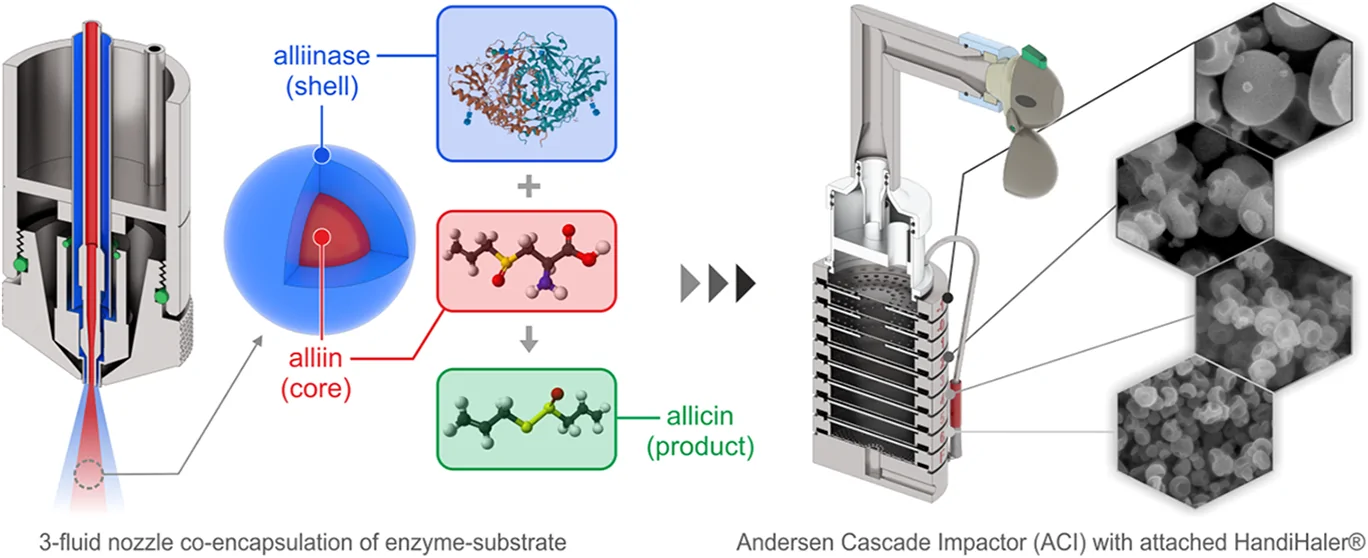

## 1 Introduction

The therapeutic properties of garlic (*Allium sativum*) have been recognized since 2000 BC, making it one of the world’s oldest known herbal medicines.^1^ The compound responsible for garlic’s distinct aroma and potency, allicin, was isolated by Chester John Cavallito and John Hays Bailey in 1944.^2^ Its molecular structure was later analyzed by Arthur Stoll and Ewald Seebeck in 1948.^3^

Despite its potential, the reactivity and short half-life of allicin present significant challenges to its practical therapeutic use. Nevertheless, the trade-off of reduced stability that results in limited exposure may be justified, as evidence suggests that the development of bacterial resistance to allicin is 1,000 times more difficult compared to β-lactam antibiotics.^4^ Furthermore, allicin targets various essential primitive bacterial functions, leaving minimal opportunity for mutation or metabolic adaptation.^5,6^ As a result, several clinically important antibiotic-resistant bacterial strains, such as methicillin-resistant *Staphylococcus aureus* (MRSA) or multidrug-resistant enterotoxigenic strains of *Escherichia coli*, *Enterococcus*, and *Shigella*, have been shown to be susceptible to allicin.^7^ Allicin has also demonstrated a significant synergistic antibacterial effect when used in combination with some conventional antibiotics, including levofloxacin, ciprofloxacin, and azithromycin.^8^

In the antibiotic era, lung pathogens continue to present a major threat because of the emergence of resistance to commonly used antibiotics and antimycotics. This situation can be particularly critical for immunosuppressed or multimorbid patients. Allicin’s bactericidal effect was confirmed against pulmonary pathogens such as *Streptococcus*, *Pseudomonas*, and *Staphylococcus*, relieving lung inflammation^9,10^ and various drug-resistant variants of *Mycobacterium tuberculosis*.^11^ Allicin’s anti-inflammatory and antibacterial properties make it a promising therapeutic agent for lung fibrosis,^12^ particularly in cases where bacterial infections such as MRSA lead to increased morbidity and accelerated mortality.^13^ Recently, allicin was found to promote airway surface liquid (ASL) secretion in murine models, suggesting its potential application as a secretory and expectorant agent for the treatment of ASL dehydration and mucus defect-associated pulmonary diseases.^14^ There is evidence from animal studies suggesting that allicin may reduce airway inflammation and remodeling,^15^ making it a potential candidate for the therapy and management of asthma^16^ and pulmonary arterial hypertension (PAH).^17^ While interest in allicin and its potential benefits is increasing, further clinical studies are necessary to validate its effectiveness for human inhalation therapy.

On the basis of these findings, pulmonary delivery of allicin offers a highly promising alternative to traditional medications for addressing the diverse respiratory disorders or issues posed by lung pathogens. However, direct inhalation of allicin in the form of vapors or nebulized aerosols may lead to excessive respiratory irritation or tissue burns, as higher allicin concentrations are required to offset dilution in the respiratory tract. In contrast, a bioinspired strategy involving the gradual *in situ* synthesis of allicin from inhaled stable precursors can achieve higher localized allicin concentrations over an extended period, thereby minimizing exposure associated with direct inhalation.

Previous studies have shown that the Büchi Mini Spray Dryer B-290 can be used to encapsulate allicin natural precursors, i.e., alliinase (enzyme) and alliin (substrate), in lactose, chitosan, TPP-chitosan, and maltodextrin microcarriers.^18–20^ Nevertheless, there is a significant, yet often overlooked, drawback. Effective in situ synthesis of allicin strictly requires the alliin and alliinase microparticles to deposit in close proximity. However, inhaling a physical mixture of these two carriers results in extensive spatial dilution; as the particles scatter across a lung surface area exceeding 70 m^2^, the probability of their codeposition decreases with every bifurcation of the bronchial tree. This poses a challenge, as the particle concentration in the airway lumen decreases rapidly from the trachea to the deep lungs (23 bifurcations in the human tracheobronchial tree resulting in 8.4 million unique routes to alveolar sacs), and only a limited amount of powder can be administered due to device constraints or patient and physiological factors. Additionally, even slight variations in the aerodynamic properties of both carrier types can result in significantly different spatial distributions within the respiratory tract.^21^

In this study, we utilized a new generation of a widely used laboratory spray dryer, the Mini Spray Dryer S-300 (Büchi), equipped with a three-fluid nozzle. Our findings demonstrate that the enzyme–substrate system can be effectively coencapsulated within micron-sized carriers without the risk of premature allicin formation. Furthermore, the allicin yields obtained upon rehydration of the microparticles were comparable to those from binary particle mixtures, with the identical alliin and alliinase loadings, prepared using a more conventional two-fluid nozzle.

First, we identified suitable spray-drying conditions and parameters for producing inhalable maltodextrin microparticles, focusing on both aerodynamic diameter and aerosolization properties. We assessed particle aerosolization properties, total emitted dose (TED), fine particle fraction (FPF), and mass median aerodynamic diameter (MMAD) with geometric standard deviation (GSD) using the HandiHaler dry powder inhaler (DPI) and the 8-stage Andersen Cascade Impactor. Next, we investigated the influence of process parameters on alliinase activity (with the two-fluid nozzle) and allicin yield (with the three-fluid nozzle) and conducted long-term stability studies under controlled conditions. Finally, we compared the particles produced by both types of nozzles for their antibacterial activity against *E. coli* and cytotoxicity toward A549 cells. To the best of our knowledge, this is the first successful preparation of inhalable powders that mimic garlic cell compartmentalization and contain both the enzyme and substrate within a single carrier.

## 2. Materials and Methods

### 2.1 Materials

Maltodextrin (dextrose equivalent 4.0–7.0), phosphate-buffered saline (PBS; tablet), pyridoxal 5′-phosphate hydrate (PLP; ≥ 98% purity), kanamycin sulfate, agar, Tricine (≥99% purity), and l-leucine were purchased from Merck. 4-Mercaptopyridine was purchased from Apollo Scientific. Ammonium carbonate and UV–vis ethanol were purchased from Lachner. Sodium chloride, tryptone, and yeast extract were purchased from Penta Chemicals. L-lactate dehydrogenase (LDH) from rabbit muscle and nicotinamide adenine dinucleotide disodium salt grade II (NADH; purity ∼ 98%) were purchased from Roche Diagnostics. Alliinase was isolated from locally sourced, fresh garlic, following the established protocol.^22^ Alliin (racemic mixture) was synthesized by Dr. Radek Jurok (the Department of Organic Chemistry, UCT Prague). *Escherichia coli* K12 (EC43) was kindly provided by Dr. Zdeněk Knejzlík (Czech Academy of Sciences).

### 2.2 Particle Preparation

#### 2.2.1 Matrix-Type Particles Prepared by a Two-Fluid Nozzle

The inhalable maltodextrin powders of matrix type (uniform composition) were prepared using Büchi Mini Spray Dryer S-300 (Figure 1A) equipped with the two-fluid (2F) kinetic nozzle (cat. No. 044698). The spray-drying addon developed by Klimša et al.,^23^ was used to accelerate the continuous preparation of powders under different process conditions. An initial full-factorial screening of selected operating parameters: (i) the inlet temperature of the drying air (100 to 200 °C), (ii) the flow rate of the atomizing air (700 to 1700 L·h^–1^), and (iii) the polymer concentration in the feed (1 to 6% w/w), allowed us to determine robust spray-drying settings that resulted in powders with suitable particle size distribution (PSD) and good aerosolization performance in combination with the DPI inhaler HandiHaler (following methodology as described in Section 2.5).

**Figure 1:**
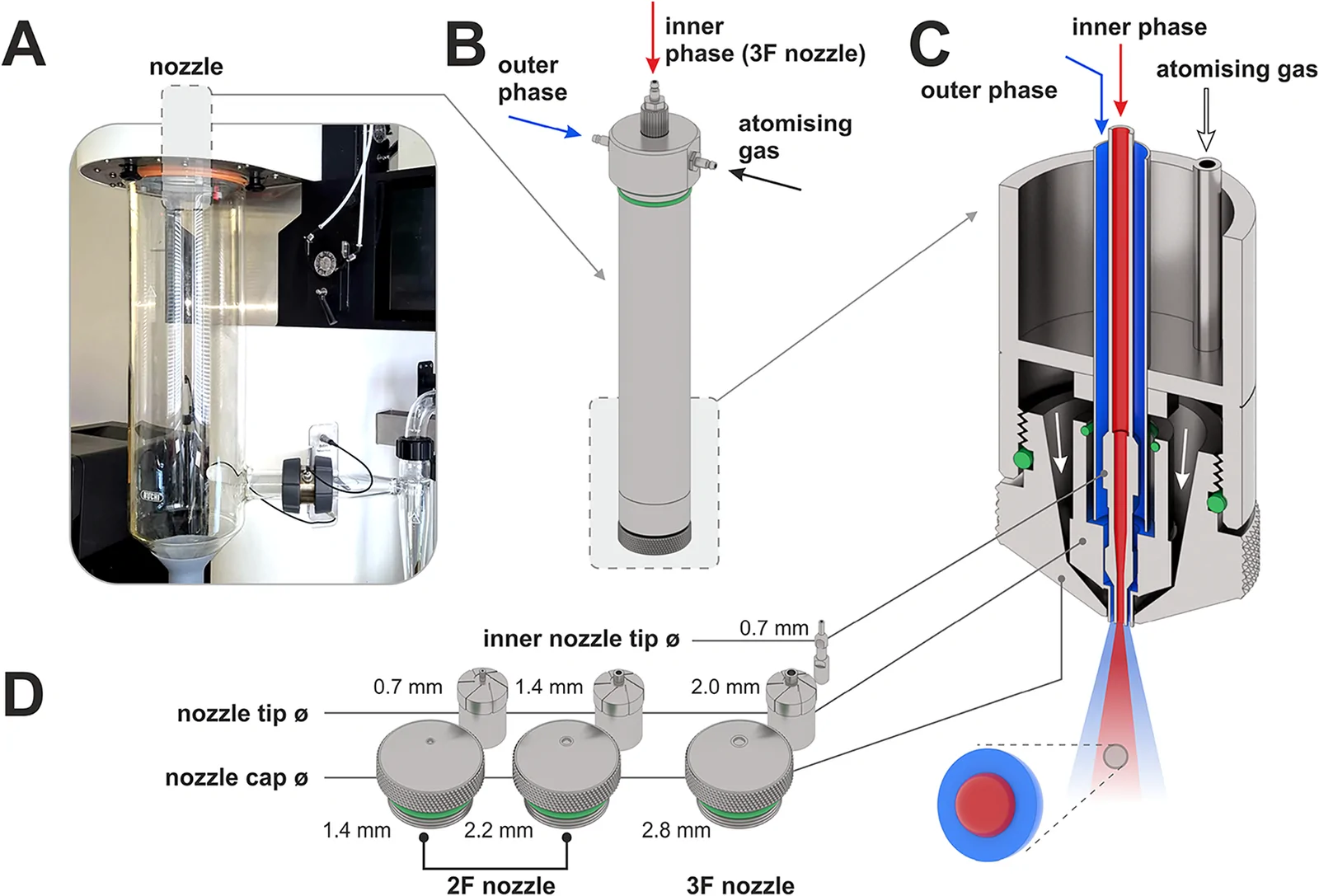
(A) Büchi Mini Spray Dryer S-300; (B) 3F nozzle with highlighted inlets of all working fluids; (C) cross-section of 3F nozzle (note: the nozzle cleaning assembly is present in 2F nozzle instead of inner phase tube); (D) nozzle caps and nozzle tip sets used in this work with 2F and 3F nozzle.

The spray-drying conditions were set as 3% *w/w* maltodextrin aqueous solution, feed flow rate: 5 mL·min^–1^, atomizing gas flow rate 1600 L·h^–1^, drying air inlet temperature 120 °C, and flow rate of drying air 20 m^3^·h^–1^. The spray-drying duration per sample (∼1 g; spray-drying product yield ≥ 60%) was typically 10 min, and the outlet temperature of the produced powder did not exceed 50 °C. The collected powder was stored in a desiccator under vacuum at room temperature for 1 day before analysis or further use. To compare the influence of 2F nozzle geometry on spray formation and PSD, two sets of nozzle caps and nozzle tips were employed: (a) 0.7 mm/1.4 mm (default) and (b) 1.4 mm/2.2 mm (Figure 1D). The composition of all particle types produced by the 2F nozzle is summarized in Table 1.

**Table 1:** Particle Types Produced by the 2F Nozzle^a^

type	description
1	maltodextrin particles with 4-MP (*W*_4‑MP_ = *m*_4‑MP_/*m*_maltodextrin_ = 1%)
2	maltodextrin particles with 4-MP and additives (various amounts of additives expressed as *w*_additives_ = *m*_additives_/*m*_dry_basis_)
3	best-performing formulations of Type 1 and 2 (in terms of aerodynamic behavior) containing substrate (alliin) or enzyme (alliinase) without 4-MP (expressed as *W*_alliin_ = *m*_alliin_/*m*_maltodextrin_ or *W*_alliinase_ = *m*_alliinase_/*m*_maltodextrin_)

a Note: *W_x_* relative mass fraction of substance *x*, *w_x_* – absolute mass fraction of substance *x*.

Particle types 1 and 2 were prepared to study the effects of nozzle geometry and additives on PSD, particle morphology, and aerodynamic performance. Type 3 was used to evaluate enzyme activity under controlled storage conditions (RH and temperature) and as a benchmark for comparison with core–shell particles prepared with the 3F nozzle in antibacterial and cell viability assays.

For example, Type 3 particles containing alliinase and maltodextrin in a mass ratio of 1:100 were prepared as follows: 3 g of maltodextrin was dissolved in 98 mL of deionized water. Subsequently, 2 mL of alliinase solution (15 mg·mL^–1^, Tric-KOH buffer 0.2 M) was added to the carrier solution to achieve the desired theoretical alliinase content (*W*_alliinase_ = 1%) in the final dried powder. The prepared solution was pumped at a constant flow rate (5 mL·min^–1^) into the spray dryer operating under steady-state conditions (*T*_in_ = 120 °C, *T*_out_ = 50 °C), atomized by a 2F nozzle, air-dried, and separated by a high-performance cyclone in a collection vessel.

#### 2.2.2 Core–Shell Particles Prepared by Three-Fluid Nozzle

To accommodate both the enzyme and the substrate within a single maltodextrin microcarrier, the three-fluid (3F) nozzle (cat. No. 11056971) and Mini Spray Dryer S-300 (Büchi) were employed. The dimensions of the 3F nozzle were as follows: inner nozzle tip ⌀ 0.7 mm, outer nozzle tip ⌀ 2.0 mm, and nozzle cap ⌀ 2.8 mm. The 3F nozzle enables the simultaneous drying of two liquid feeds (Figure 1B), which are transported separately to the nozzle orifice, where they form a spray (Figure 1C). Therefore, the time during which the two liquids are in physical contact is limited to the lifespan of the liquid droplet, which is typically less than 1.5 s in the Büchi S-300 spray drier. Such short contact time is expected to prevent the formation and excessive loss of allicin during the drying process. However, because the 3F nozzle enables adjusting flow rate ratios, the order of atomized fluids, and their composition, various combinations were tested to (i) minimize the unwanted formation of allicin during spray drying, and (ii) achieve complete alliin conversion upon particle dissolution. In most cases, the inner phase contained dissolved substrate alliin, while the enzyme was present in the outer phase. In an idealized case, particles produced by a 3F nozzle should have a core–shell structure, with the substrate located in the core and the enzyme in the shell (in contrast to the uniform composition of matrix-type particles). The spray-drying operating parameters used with the 2F nozzle remained unchanged across all experiments (see Table 2). Similar to the 2F nozzle, two types of particles (without and with additives) containing 4-MP (*W*_4‑MP_ = 1%) were prepared to evaluate the effects of the 3F nozzle geometry and additives on PSD, particle morphology, and aerodynamic performance. To investigate the relationship between allicin yield and operating parameters specific to the 3F nozzle, the following Type 3 particles were prepared:

i) Various alliinase:alliin mass ratios - The volumetric ratio of both phases (2.5:2.5 in mL·min^–1^) was fixed, alliin particle content was kept constant at *W*_alliin_ = 1%, and alliinase:alliin mass ratio varied from 1:1, 1:10, to 1:100.
ii) Various total alliin amount - The volumetric ratio of both phases (2.5:2.5 in mL·min^–1^) was fixed, the total alliin amount was varied to achieve *W*_alliin_ = 1, 2, and 4%, and the alliinase:alliin mass ratio was 1:10.
iii) Various flow rate ratios - The volumetric flow rate ratio of inner and outer liquid was varied (0.5:4.5, 1.5:3.5, 2.5:2.5, 3.5:1.5, and 4.5:0.5 in mL·min^–1^). The alliin concentration in the inner phase was varied to give *W*_alliin_ = 1%, and the alliinase:alliin mass ratio in the final product was fixed to 1:10.
iv) Phase inversion – The volumetric ratio of both phases (2.5:2.5 in mL·min^–1^) was fixed, alliin particle content was kept constant at *W*_alliin_ = 1%, and the alliinase:alliin mass ratio was 1:10. In contrast to previous settings, alliinase was present in the inner phase (core) and alliin in the outer phase (shell).

**Table 2:** Mean Particle Size, TED, FPF, MMAD, and GSD for All Tested Combinations of Compositions and Nozzle Types^a^,^b^,^c^

sample	nozzle	carrier material	mean size [μm]	TED [%]	FPF [%]	MMAD [μm]	GSD
1	2F	Maltodextrin	2.5 ± 0.3	72 ± 28	17 ± 2.0	3.6 ± 0.5	2.0 ± 0.1
2	2F^b^	Maltodextrin	9.2 ± 1.1	99 ± 2	9 ± 0.5	6.0 ± 0.5	1.9 ± 0.0
3	3F	Maltodextrin	4.0 ± 0.1	98 ± 3	13 ± 3.0	3.6 ± 0.6	2.5 ± 0.2
4	2F	Maltodextrin +1% porogen	2.7 ± 0.4	85 ± 2	21 ± 2.0	3.6 ± 0.2	1.9 ± 0.0
5	2F	Maltodextrin +10% porogen	3.9 ± 0.4	58 ± 27	22 ± 2.0	3.4 ± 0.2	1.9 ± 0.1
6	2F	Maltodextrin +30% porogen	3.7 ± 1.2	99 ± 2	15 ± 3.0	3.4 ± 0.1	1.9 ± 0.0
7	2F	Maltodextrin +5% leucine	2.5 ± 0.2	94 ± 6	18 ± 4.0	3.2 ± 0.6	2.0 ± 0.2
8	2F	Maltodextrin +10% leucine	2.3 ± 0.1	98 ± 3	36 ± 1.0	3.6 ± 0.1	1.8 ± 0.0
9	2F	Maltodextrin +15% leucine	2.4 ± 0.1	95 ± 9	18 ± 7.0	2.9 ± 0.8	2.2 ± 0.4
10	3F	Maltodextrin +1% porogen	3.4 ± 0.6	91 ± 15	19 ± 4.0	2.8 ± 0.3	2.2 ± 0.0
11	3F	Maltodextrin +10% porogen	3.0 ± 0.5	82 ± 22	22 ± 3.0	3.9 ± 0.5	1.9 ± 0.1
12	3F	Maltodextrin +30% porogen	2.9 ± 0.2	88 ± 13	23 ± 3.0	3.8 ± 0.3	1.9 ± 0.1
13	3F	Maltodextrin +5% leucine	4.0 ± 0.3	100 ± 0	33 ± 0.3	4.0 ± 0.1	1.8 ± 0.0
14	3F	Maltodextrin +10% leucine	3.6 ± 0.2	99 ± 1	34 ± 8.0	3.8 ± 0.4	1.9 ± 0.2
15	3F	Maltodextrin +15% leucine	3.4 ± 0.1	99 ± 2	29 ± 3.0	3.7 ± 0.1	1.9 ± 0.0

a Data are presented as Mean ± SD, sample size (*n* = 3).

b 2F nozzle with a larger nozzle tip ⌀ 1.4 mm and nozzle cap ⌀ 2.2 mm.

c Spray-drying conditions: inlet temperature – 120 °C, atomizing gas flow rate – 1600 L·h^–1^, total flow rate – 5 mL·min^–1^, 3 w/w % maltodextrin as carrier solution. Stock setup: 2F nozzle with nozzle tip ⌀ 0.7 mm, nozzle cap ⌀ 1.4 mm. 3F nozzle with inner nozzle tip ⌀ 0.7 mm, outer nozzle tip ⌀ 2.0 mm, nozzle cap ⌀ 2.8 mm.

### 2.3 Choice of Additives

To enhance aerosolization and handling characteristics of spray-dried maltodextrin particles produced by 2F and 3F nozzles (Type 1), the use of two additives was explored (Type 2 particles). Ammonium carbonate was selected as a porogen due to its thermal decomposition into carbon dioxide and ammonia at temperatures exceeding 60 °C. These morphological changes, resulting from the formation of gas bubbles, have been reported to reduce particle density and improve aerodynamic properties.^24^ Next, leucine was selected as a surface-active compound. Leucine, when spray-dried from aqueous feedstocks, predominantly accumulates on the particle surface interface with the hydrophobic tail facing outward. There, it quickly reaches supersaturation and forms a crystalline hydrophobic outer shell, which shrivels, resulting in hollow wrinkled particles with a hydrophobic surface. Leucine-modified particles were expected to exhibit improved aerodynamic properties, enhanced powder flowability and dispersibility, and reduced moisture-induced degradation of enzyme and substrate over time.^25,26^ In the case of the 3F nozzle, the presence of leucine was expected to minimize moisture uptake and spontaneous enzymatic formation of allicin during storage.

### 2.4 Particle Size and Morphology

The particle size distribution (PSD) was analyzed by using a static light scattering (SLS) method in wet mode (Horiba Partica LA-950 S2). Before measuring the PSD, particles were dispersed in absolute ethanol and sonicated for 5 min to break apart temporary agglomerates. The particle suspension was then added to a quartz sample cell prefilled with absolute ethanol under constant stirring. All measurements were performed in triplicate.

A scanning transmission electron microscope (SEM), Jeol JCM-5700, was used to observe the particle size and surface morphology. A powder sample was attached to a carbon conductive tape and dusted off with compressed gas, and the surface was sputter-coated with a 5 nm gold layer (Emitech K550X) to prevent sample charging and drift during image acquisition. All measurements were performed at an acceleration voltage of 5 to 10 kV.

### 2.5 Aerodynamic Behavior of Particles

Powders intended for use in dry powder inhalers must have favorable aerodynamic properties to allow deep lung deposition. Generally, this requires a combination of small particle size and low particle density. All of these factors are combined in the so-called aerodynamic diameter, i.e., the diameter of a sphere with water density and the same terminal settling velocity as the given particle.^27^ Therefore, the prepared powders were tested primarily for their PSD and aerodynamic behavior. The latter is assessed using the total emitted dose (TED) and fine particle fraction (FPF). TED stands for the total amount of powder emitted from the loaded capsule. FPF expresses the fraction of the emitted powder with the aerodynamic diameter smaller than 5 μm, i.e., particle size usable for deep lung delivery. The aerodynamic performance of powders was also critically evaluated using the Mass Median Aerodynamic Diameter (MMAD) and Geometric Standard Deviation (GSD), which respectively predict the primary site of airway deposition and quantify aerodynamic polydispersity. Homogeneous formulations suitable for deep pulmonary delivery typically require an MMAD between 1 and 5 μm (restricted to 1–3 μm for purely alveolar targeting) coupled with a GSD of <2.0 (optimally ≈ 1.5) to maximize the fine particle fraction. All these parameters are relevant for *in vitro–in vivo* correlations.^28^

The behavior of powders emitted from a commercial dry powder inhaler (DPI, HandiHaler) was assessed using ACI, i.e., 8-stage Andersen Cascade Impactor (TCR-Tecora) equipped with a preseparator and a USP induction port (Figure 2). During the experiment, a constant airflow rate of 60 L min^–1^ was set in the ACI using an external pump with an automatic flow control (Bravo X H Bio).

**Figure 2:**
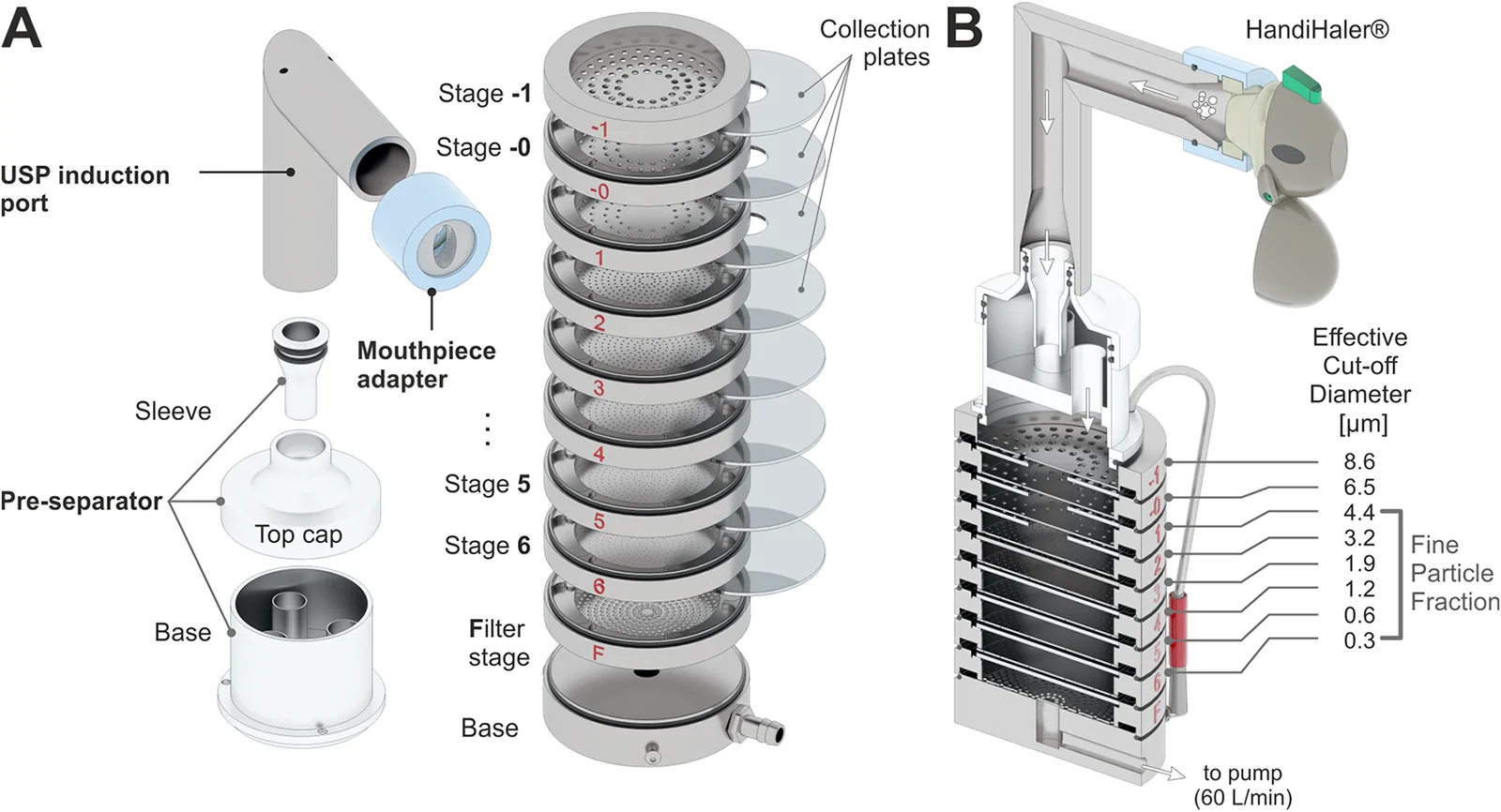
(A) Exploded diagram of 8-stage ACI with 60 L·min^–1^ conversion kit; (B) cross-section of ACI with attached DPI (HandiHaler) and *d*_50_ values for stages no. −1 to 6.

The powder (approximately 30 mg, for compositions see Table 2) was gently loaded into a hard gelatin capsule (size 3, Noventis), placed into the DPI, perforated as instructed by the HandiHaler manual, and pressed against the silicon mouthpiece of the ACI. The HandiHaler was left in place for 5 s to allow powder to be emitted, and the used capsule was checked for residual powder. Two capsules with the same powder formulation were used for a single measurement to ensure sufficient powder deposition across all ACI stages, enabling reliable UV–vis quantitative analysis of 4-MP content. Particles collected on stages 1 to 6 were used to calculate FPF (cutoff diameter 4.4 to 0.3 μm at 60 L·min^–1^). This setup, with a specified airflow rate and commercial DPI, aims to replicate real inhalation drug-delivery conditions as closely as possible. Next, the ACI collection plates with deposited particles were submerged in 10 mL of demineralized water to dissolve all adhered powder. Then, the absorbance of the solution was measured at 324 nm (absorbance maximum of 4-MP) using a WPA Lightwave II spectrophotometer.

It is important to note an *in vitro* limitation regarding the aerodynamic testing setup. In this study, the HandiHaler was operated at a constant flow rate of 60 L·min^–1^ to ensure capsule emptying and to provide a consistent deagglomeration energy. This approach was chosen to minimize device-related retention variability, thereby allowing for a robust fundamental comparison of the inherent aerodynamic properties (i.e., FPF, MMAD, and GSD) across all tested powder formulations. While drawing air through the high-resistance HandiHaler at 60 L·min^–1^ generates a pressure drop that exceeds the pharmacopeial standard of 4 kPa, clinical data indicate that patients with moderate to very severe COPD can still achieve peak inspiratory flow rates of approximately 46–50 L·min^–1^ through this device.^29^ Furthermore, established literature demonstrates that the aerosolization efficiency and delivered dose of the HandiHaler remain relatively constant and largely flow-independent between 30 and 60 L·min^–1^.^30,31^ Nevertheless, the standardized constant flow rate used herein represents an *in vitro* condition. As demonstrated by Mišík et al.,^32^ device pressure drop heavily influences realistic patient breathing profiles and subsequent aerosol deposition in human airways. Therefore, the aerodynamic parameters reported in this study reflect the comparative *in vitro* deagglomeration potential of prepared powders rather than their direct *in vivo* clinical deposition.

### 2.6 Allicin Content Evaluation (4-MP assay)

The amount of enzymatically produced allicin after the dissolution of spray-dried maltodextrin powders was assessed spectrophotometrically by assessing its reaction with 4-mercaptopyridine.^33^ Since the resulting product, 4-allylmercaptothiopyridine, does not absorb at 324 nm, the initial reaction rate can be represented as the negative slope of the decrease in absorbance over time. The initial reaction rate depends on the allicin concentration; therefore, a calibration curve can be used to assess the allicin content in the powders.

The reaction mixture contained 34 μM 4-MP and 13 to 260 μM allicin dissolved in PBS. The reaction was monitored for 10 min, and the slope was assessed for the linear portion of the absorbance–time curve. The calibration curve (quadratic polynomial, *R* ^2^ = 0.995) was determined using allicin samples prepared from a pure alliin/alliinase mixture, assuming complete alliin conversion, with an alliinase concentration of 0.08 mg·mL^–1^. The appropriate amounts of tested powders were dissolved in PBS buffer and measured after 60 min. This duration and enzyme concentration were found to be sufficient for the complete conversion of alliin to allicin. The absorbance measurements were conducted using a UV–vis spectrophotometer SPECORD 205.

### 2.7 Testing of Enzyme Activity

The alliinase was extracted from peeled fresh garlic following the protocol of Mallika et al., with some minor modifications.^22,34^ The alliinase’s protein content was determined by Bradford assay, and specific enzyme activity was assessed using the L-lactate dehydrogenase (LDH) assay as previously described by Mašková et al.^20^ Here, the byproduct of allicin production, pyruvate, is reduced to lactate using the LDH enzyme, while the cofactor NADH is oxidized to NAD^+^, resulting in a significant decrease in absorbance at 340 nm. The concentrations of reactants used in the assay were as follows: alliin (200 mM), NADH (0.8 mM), PLP (0.02 mM), LDH (0.25 *v/v* %), and alliinase (lyophilized or spray-dried in concentration 12.5 μg·mL^–1^) in a total reaction volume of 100 μL. These specific conditions were chosen to ensure zero-order kinetics, which is essential for the reliable determination of enzyme activity. All measurements were conducted in triplicate.

### 2.8 Effect of Relative Humidity

The impact of relative humidity (RH) on the powder storage was tested using sealed chambers containing saturated salt solutions to create different relative humidities: 33% RH (MgCl_2_), 53% RH (Mg­(NO_3_)_2_), 75% RH (NaCl), and 100% RH (demineralized water). The powders were spread into thin layers, placed in humidity chambers, and stored in an incubator set to a constant temperature of 25 °C for up to one month. The 2F and 3F samples were characterized at specified times for remaining enzyme activity and allicin content, respectively. Each data point and RH combination had a designated storage chamber, ensuring that the controlled atmosphere remained undisturbed throughout the entire experiment. Initial and equilibrium moisture contents were determined gravimetrically, and all measurements were performed in triplicate.

### 2.9 Antibacterial Activity

#### 2.9.1 Bacteria Cultivation

In our previous work, we showed that *in situ*-formed allicin (in liquid and gas phase) has a comparable antibacterial effect on Gram-negative and Gram-positive bacterial strains, including *S. aureus*.^19^ Therefore, all testing in this work was performed, for simplicity, using only Gram-negative *E. coli*. The bacteria were cultivated in Luria–Bertani (LB) medium, prepared as follows: 10 g Tryptone, 5 g yeast extract, and 5 g NaCl were dissolved in 1 L of demineralized water, and the pH was adjusted to 7.2–7.4 with 1 M sodium hydroxide solution. 15 g of agar was added to the LB medium to prepare agar plates. Bacterial colonies were resuspended in 10 mL of LB medium and grown overnight at 37 °C. Prior to the experiments, 1 mL of the bacterial suspension was added to 6 mL of LB medium and cultivated at 37 °C until OD_600_ reached 0.4. Finally, 150 μL of this suspension was spread onto the surface of the LB-agar plate.

#### 2.9.2 Antibacterial Properties of Prepared Powders

The antibacterial properties of the prepared powders were tested using two methods: the standard disk diffusion method and a contactless assay. The well-established disk diffusion test was used to demonstrate allicin’s antibacterial activity in the liquid phase. First, a given amount of particles containing alliin and alliinase was dissolved in PBS and left to react for one hour, which was sufficient to assume complete conversion of alliin to allicin. Then, 20 μL of the solution was applied to a small disk of filtration paper placed directly on the agar plate inoculated with bacteria. Commercially available antibiotic kanamycin (50 mg·mL^–1^, 10 μL) served as a positive control, while PBS (10 μL) was used as a negative control. The agar plates were incubated overnight at 37 °C, and the inhibition zones were then observed. All experiments were conducted in triplicate.

The contactless assay was used to investigate the antibacterial effect of allicin vapors generated by the particles, without direct contact between the powder and the bacteria. Up to 20 mg of powder was placed in a 3D-printed sample holder, covered with a dialysis membrane (MEMBRA-CEL, MWCO 14 kDa), and secured with a retaining ring. The holder was then placed on the agar plate, leaving a gap between the sample and the inoculated surface. The positive control remained the same as in the disk diffusion test, with a kanamycin solution applied to the filtration paper disk. As a negative control, 10 μL of the same kanamycin solution was applied to the 3D-printed holder. Since the dialysis membrane is permeable only for vapors, the nonvolatile kanamycin should have no antibacterial effect in the contactless assay. The agar plates were incubated overnight at 37 °C, and the inhibition zones emerging under the footprint of the holder were evaluated. All experiments were done in triplicate.

### 2.10 Cell Viability

#### 2.10.1 Cells and Media

The A549 (human, epithelial, lung, disease: pulmonary adenocarcinoma) cell line purchased from CLS Cell Lines Service GmbH was cultured in Dulbecco’s Modified Eagle’s Medium-high glucose (DMEM; Merck D6429) supplemented with 10% heat-inactivated fetal bovine serum (Merck F7524), 1% nonessential amino acids (Merck M7145), and 1% Antibiotic-Antimycotic Solution (Merck A5955). Cells reaching 70–80% confluency were subcultured using a 0.25% trypsin–EDTA solution and incubated at 37 °C in 5% CO_2_.

#### 2.10.2 Cell Viability Assay

A549 cells were harvested and counted using a Bürker counting chamber in the presence of Trypan Blue. The cell suspension was diluted in fresh culture medium to a concentration of 10^5^ cells·mL^–1^, and 100 μL was seeded per well, corresponding to 10^4^ cells per well. Cells were seeded into transparent, flat-bottom 96-well culture plates and allowed to adhere and acclimate for 24 h in a humidified incubator (5% CO_2_, 37 °C).

To prevent undesired cell detachment during washing, the original seeding medium was retained in the wells. Subsequently, 100 μL of the test samples prediluted in the culture medium was added to each well, bringing the total volume to 200 μL. Sixteen wells served as controls, i.e., 8 wells were left untreated (positive control, 100% viability), and 8 wells were later treated with 1% Triton X-100 (negative control, 0% viability). The cytotoxic effect of *in situ*-formed allicin was evaluated in A549 cells over a concentration range of 0.78–100 μg·mL^–1^. Allicin was formed from (i) 1:1 powder blend of *W*_alliin_ = 2.0% and *W*_alliinase_ = 0.2% 2F maltodextrin particles, and (ii) 3F particles containing both alliin and alliinase (*W*_alliin_ = 1.0%, *W*_alliinase_ = 0.1%). Physical mixture of 2F particles and 3F particles was comparable in terms of absolute composition and theoretical allicin content. As controls, alliin-maltodextrin particles (*W*_alliin_ = 2.0%), alliinase-maltodextrin particles (*W*_alliinase_ = 0.2%), and pure maltodextrin particles were tested. The mass of each sample corresponded to the amount required to theoretically generate a given allicin concentration (e.g., 100 μg·mL^–1^), assuming 100% conversion. Plates containing each formulation type, i.e., powder blends and single particles, were incubated for either 1 or 4 h. After the designated exposure time, the medium was removed from all wells by gentle aspiration, replaced with 100 μL of fresh culture medium, and incubated to complete the 24-h exposure period.

After 24 h, 1% *w/v* Triton X-100 was added to 8 wells to lyse the cells (negative control, 0% viability), while the other 8 control wells remained untreated (positive control, 100% viability). After 10 min, all media were gently removed by aspiration, and 100 μL of 0.1 mg·mL^–1^ resazurin solution was added to each well. Viable cells reduce resazurin to the fluorescent compound resorufin (λ_ex_ = 560 nm, λ_em_ = 590 nm), enabling viability assessment using a calibration curve prepared on the same plate with dedicated controls. After one hour of incubation, fluorescence was measured using a Tecan Infinite M200 plate reader operated by i-control software. IC_50_ values were calculated using the IC50 Calculator (AAT Bioquest).

## 3 Results and Discussion

### 3.1 Particle Characterization

The following section systematically examines the influence of nozzle type and geometry, as well as the incorporation of specific additives such as ammonium carbonate (employed as a porogen) and leucine (functioning as a surface modifier and dispersibility enhancer), on the aerodynamic characteristics of maltodextrin powders. A comprehensive summary of the results is presented in Table 2. The tested formulations always contained 4-MP (*W*_4‑MP_ = 1%), permitting highly sensitive UV–vis quantification. Preliminary comparison of spray-dried maltodextrin particles without and with added 4-MP showed no observable difference in particle PSD, morphology, handling, or aerosolization performance.

#### 3.1.1 Nozzle Types

Thanks to the initial full-factorial screening of operating parameters, the mean particle size of pure maltodextrin particles prepared by 2F and 3F nozzles did not exceed 2.5 and 4.0 μm, respectively (ref samples 1 and 3 in Table 2). When the 2F nozzle was modified with a larger nozzle tip and cap, the mean particle size was almost 4 times larger, and the MMAD exceeded 5 μm, making this sample unsuitable for pulmonary delivery (sample 2). The total emitted dose (TED) was low and highly variable for the finer particles produced by the 2F nozzle (72 ± 28%). Larger particles (samples 2 and 3) consistently resulted in nearly complete powder emission from the loaded capsule, with only minor deviations. On the contrary, the fine particle fraction (FPF) was the lowest for coarse particles (sample 2). SEM images of individual fractions of 2F (sample 1) and 3F particles (sample 3) separated by 8-stage ACI according to their aerodynamic diameter are shown in Figure SI 1. The data indicate that achieving an FPF greater than 20% is a challenge for pure maltodextrin carriers. Consequently, the following section will investigate the impact of additives on improving aerosolization properties.

#### 3.1.2 The Effect of Additives

*Ammonium carbonate*. Using ammonium carbonate as a porogen results in mean size fluctuations without any clear trend, regardless of porogen concentration or nozzle type (2F nozzle – samples 4–6, 3F nozzle – samples 10–12). However, the median particle diameter (Dv50) for 3F samples is slightly reduced with increasing porogen content (Figure SI 3). The addition of ammonium carbonate gradually transformed the buckled shape of pure maltodextrin particles (Figure 3A) into undented smooth spheres (Figure 3B and Figure SI 4). Most importantly, powder handling became particularly challenging due to the presence of hard agglomerates, which complicated tasks such as sample collection, capsule loading, and routine SLS sample preparation. In some cases (samples 5 and 11), TED values were lower and more uncertain than those for pure maltodextrin particles produced by the same nozzles. On the other hand, FPF has increased above 20% (Figure SI 7). MMAD and GSD values show that all samples with added porogen were appropriate for deep lung delivery. However, the lingering ammonia smell that persisted for several weeks after preparation was another reason not to pursue the use of ammonium carbonate further. Intentionally elevating the inlet temperature to 170 °C did not show any noticeable difference in particle properties, indicating that 120 °C was sufficient for the thermal decomposition of ammonium carbonate.

**Figure 3:**
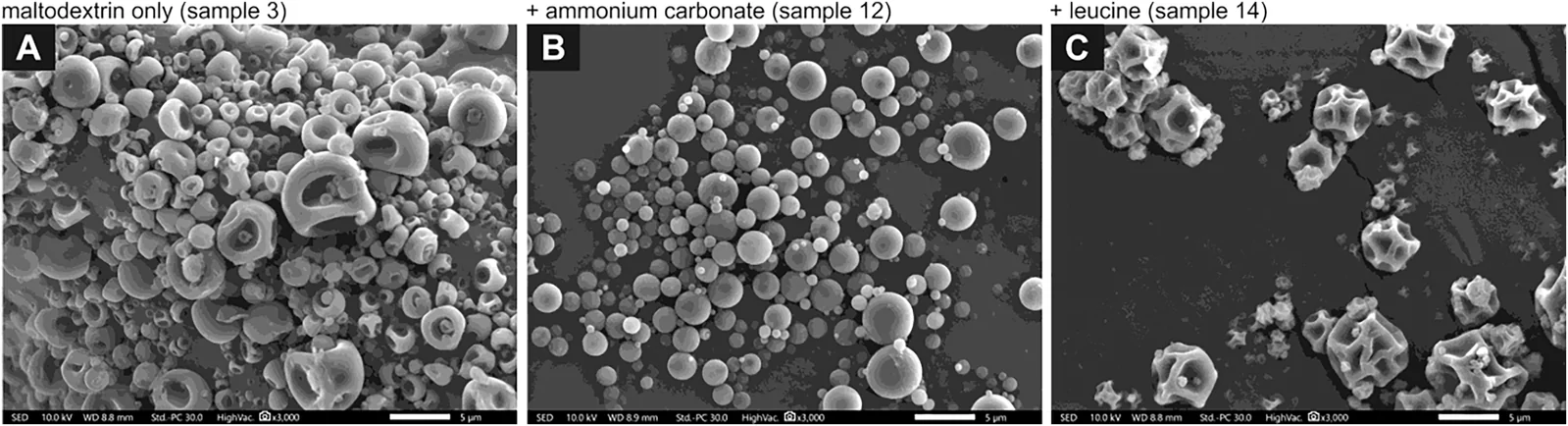
Morphology of maltodextrin 3F particles without (A) and with additives (B) – 30% *w/w* of ammonium carbonate; (C) – 10% *w/w* of leucine.

*Leucine*. In contrast to ammonium carbonate, the critical steps, i.e., spray drying, sample collection, and handling of leucine-modified maltodextrin powders, were much more straightforward, regardless of the examined leucine content or nozzle selection. For the 2F nozzle, the particle mean size and PSD were not further reduced with increasing leucine content (samples 7–9). For the 3F nozzle, increased leucine content led to a smaller mean particle size (samples 13–15), likely due to its weak surfactant properties aiding liquid atomization (Figure SI 5). In terms of particle morphology, increasing leucine content accentuated the buckling of maltodextrin particles (Figure 3C and Figure SI 6). Particles produced by both nozzles with 10% *w/w* leucine (samples 8 and 14) showed both high TED (>95%) and FPF (>30%). Furthermore, samples 8 and 14 demonstrate optimal aerodynamic profiles for deep lung delivery, evidenced by their MMAD (3.6 ± 0.1 and 3.8 ± 0.4 μm, respectively) and consistently narrow size distributions (GSD < 2.0). Based on ease of handling and ACI results (summarized in Table 2), the 10% *w/w* leucine formulation was further used for alliin and alliinase encapsulation using 2F and 3F nozzles, and for evaluation of powder stability across different levels of relative humidity.

The importance of integrating robust *in vitro* methods with *in silico* modeling to accurately predict regional deposition patterns is well-recognized.^35,36^ Applying the Multiple Path Particle Dosimetry software (MPPD v3.04, ARA, Arlington, VA, USA) to our experimental data under simulated dry powder inhalation conditions (inspiratory flow rate of 60 L·min^–1^ with a 5-s breath-hold) revealed highly favorable deposition profiles for deep lung delivery. Specifically, the *in silico* prediction for the 2F sample 8 estimated a pulmonary (alveolar) deposition of 55%, a tracheobronchial deposition of 16%, and an extrathoracic (head) deposition of 20%. For the 3F sample 14, the estimated pulmonary deposition was 50%, alongside a tracheobronchial deposition of 17% and an extrathoracic deposition of 24%. Overall, these predictive findings clearly demonstrate that the aerodynamic properties of the 10% *w/w* leucine formulation prepared by both nozzles fall well within the respirable range required to achieve a clinically relevant spatial distribution. This extensive deep lung penetration is the fundamental prerequisite for the effective *in situ* formation of allicin over the large surface area of the lower airways.

### 3.2 2F Nozzle: Alliinase’s Specific Activity Assessment

Initially, both D-mannitol and maltodextrin were considered as suitable materials for alliinase encapsulation. D-mannitol is widely used as an excipient and therapeutic agent in inhalation applications,^37^ whereas maltodextrin has been shown to be highly effective in protecting alliinase from degradation.^20^ The enzymatic activity of encapsulated alliinase (by 2F nozzle) was assessed through LDH assay after 24 h of storage in a desiccator. Results were compared with the specific activity of lyophilized alliinase that was not subjected to spray drying, which was 1.05 μM·s^–1^·mg^–1^. Consistent with our previous findings,^20^ the specific activity values for alliinase encapsulated in maltodextrin were nearly identical to those of the stock lyophilized enzyme, with relative activity exceeding 95%. The presence of leucine as an additive (up to 15% *w/w*) had no observable effect on alliinase activity in maltodextrin particles. In contrast, D-mannitol provided much lower alliinase stabilization, with the specific activity reduced to only 50%. D-mannitol particles also did not demonstrate any advantages in terms of the FPF (Figure SI 2). These findings further confirm maltodextrin’s suitability for alliinase encapsulation.

### 3.3 3F Nozzle: Coencapsulation of Alliin and Alliinase

Maltodextrin core–shell particles containing both alliin and alliinase were produced using the 3F nozzle. In this setup, preventing premature allicin formation is crucial. Therefore, the impact of different arrangements specific to the 3F nozzle (as mentioned in Section 2.2.2) was examined to assess and help to reduce allicin loss during atomization of both feeds, solvent evaporation, and particle formation. The data are presented using the following formulation as a reference: alliinase-alliin mass ratio of 1:10; alliin content *W*_alliin_ = 1%, and volumetric feed ratio of 1:1 with alliin dissolved in the inner phase (Figure SI 9). This specific formulation was confirmed to achieve the theoretical yield of allicin after rehydration as measured by the 4-MP assay.

#### 3.3.1 Enzyme to Substrate Ratio

Three different mass ratios of alliinase (variable) to alliin (fixed) were tested: 1:1, 1:10, and 1:100. Additionally, the 1:1 ratio was compared to a sample created from a physical mixture of alliinase-maltodextrin and alliin-maltodextrin particles (with the same alliinase and alliin loadings) prepared by the 2F nozzle under identical conditions. After the powders were dissolved and analyzed for allicin content, fresh alliinase was added to assess whether the amount of encapsulated alliinase affected the observed allicin yield (Figure 4).

**Figure 4:**
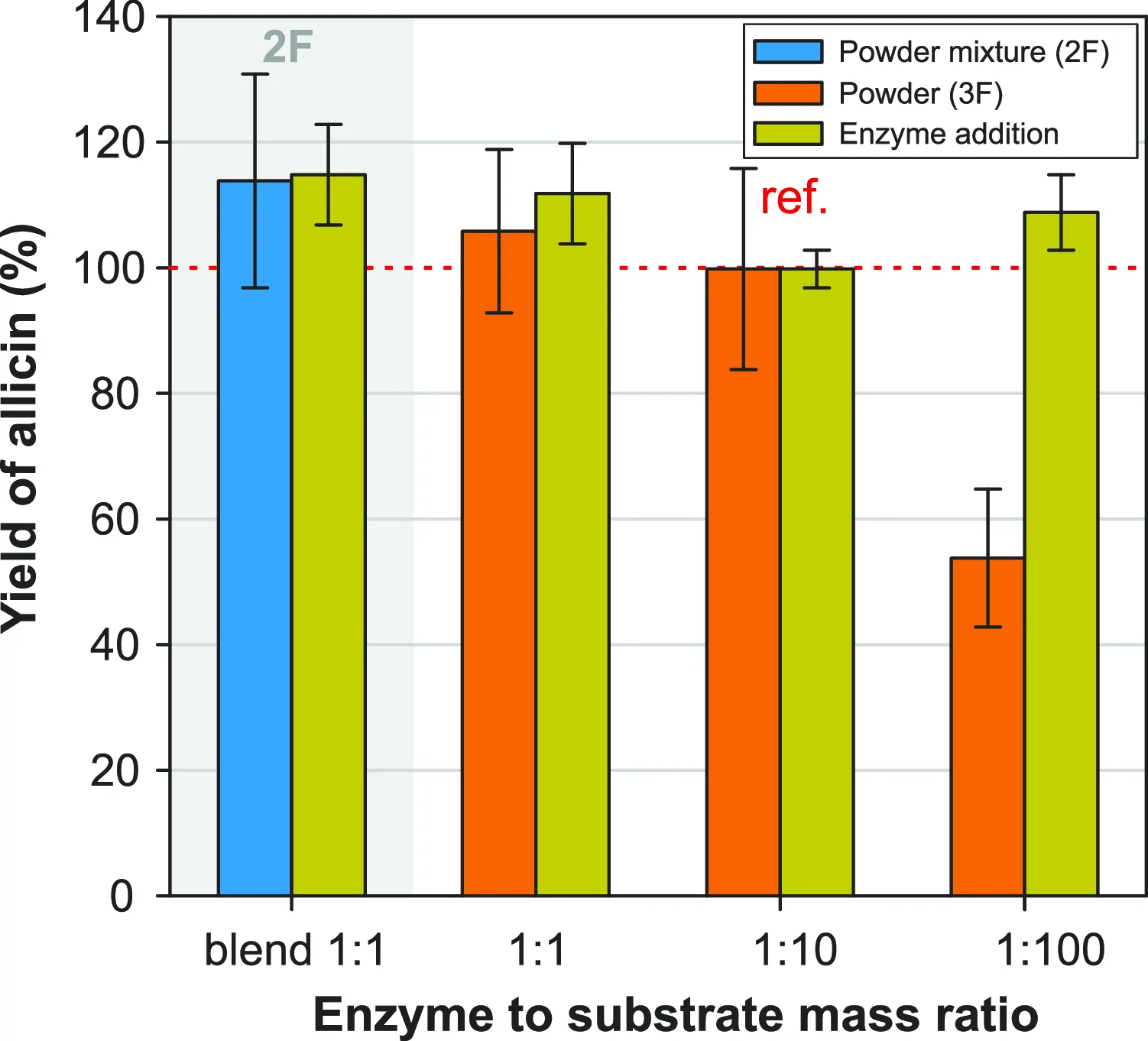
Yield of allicin vs enzyme–substrate mass ratio. Data are presented as mean ± SD, sample size (*n* = 3).

The samples with 1:1 and 1:10 mass ratios achieved allicin levels comparable to those of the 2F particle blend. These results suggest that very short contact times between the substrate and enzyme phases with the 3F nozzle do not result in a significant loss of allicin. However, when the enzyme content was further reduced to a 1:100 ratio, the encapsulated alliinase was insufficient to convert all the alliin present in the sample, resulting in a 2-fold decrease in allicin production (Tukey’s post hoc test, *p* < 0.05). Confirming this, the addition of enzyme to dissolved particles showed that once fresh alliinase was introduced, the remaining alliin was immediately converted. The 1:10 alliinase-alliin ratio was therefore chosen for further experiments, providing a high allicin yield comparable to that of the 1:1 2F particle blend and reasonable enzyme consumption. Repeated experiments with the same particles, stored for one month at −20 °C, yielded comparable results.

#### 3.3.2 Alliin Loading

Up to a 4-fold increase in alliin loading (*W*_alliin_ = 1 to 4%) at a fixed volumetric ratio, and a 1:10 alliinase to alliin mass ratio was tested to determine whether higher loadings of both alliinase and alliin result in increased reactivity within atomized fluids. Regardless of alliin loading, all formulations achieved predicted allicin yields. Consequently, the theoretical dosage of allicin in the single-particle formulation can be increased at least 4-fold, if required, without risking unwanted reactions and allicin loss during drying. After 30 days, the identical particles stored at −20 °C showed unchanged results. Given the high reagent consumption per experiment, higher alliin loadings were not further explored.

#### 3.3.3 Feed Flow Rate Ratio

Using a 3F nozzle allows for the adjustment of the flow rate ratio between the inner phase (feed_in) and outer phase (feed_out) (Figure 1C). An experiment was conducted to produce particles with a fixed theoretical final composition, while the feed_in:feed_out flow ratio varied from 0.5:4.5, 1.5:3.5, 2.5:2.5, 3.5:1.5, to 4.5:0.5, with alliin present in the inner phase. Additionally, for the 2.5:2.5 ratio, an inverse configuration was tested in which alliin was replaced with the enzyme and vice versa. The overall feed flow rate was maintained at 5 mL·min^–1^ to prevent the outlet temperature from exceeding 50 °C and thereby preventing enzyme degradation. Among the tested ratios with the enzyme in the outer phase, no statistically significant difference in allicin yield was observed after dissolution (ANOVA test, *p* > 0.05). For practical work with the 3F nozzle, a 1:1 ratio (2.5 mL·min^–1^ per phase) was preferred; however, no abnormal atomization behavior, such as pulsation or uneven spray formation, was observed for other investigated ratios. For reference, the SEM images and PSD of all samples are summarized in Figure SI 10 and Figure SI 11, respectively.

On the other hand, the inverse 1:1 formulation, with alliinase in the inner phase and alliin in the outer phase, showed consistently more than a 20% decrease in observed allicin yield compared to the regular 1:1 ratio formulation with alliinase in the outer phase (ANOVA, Tukey’s post hoc test, *p* < 0.05). These results suggest that the spatial distribution of the reactants significantly impacts the yield, a phenomenon that can be fundamentally explained using the dimensionless Péclet number (Pe). In the context of spray drying, Pe defines the ratio of the radial velocity of the receding droplet surface to the diffusion coefficient of the solute.^25^ The high-molecular-weight alliinase (*M*_w_ ∼ 90 kDa) possesses a very low diffusion coefficient, corresponding to a high Pe. Consequently, when dissolved in the outer phase, it cannot diffuse rapidly enough to keep pace with the receding air–liquid interface and preferentially accumulates at the surface. Conversely, the hydrophilic low-molecular-weight alliin (*M*_w_ = 177.22 Da) exhibits a high diffusion coefficient (low Pe), allowing it to effectively avoid entrapment at the droplet surface and migrate toward the liquid core. In the inverse scenario, the rapidly diffusing alliin from the outer phase migrates into the enzyme-rich core. Here, alliinase remains dissolved and active for a longer duration because the presence of a growing solid crust reduces the evaporation rate. As a result, more alliin is prematurely converted into allicin. The enzyme addition experiment further supports this theory, as it did not result in higher allicin yields for the inverse formulation. This indicates that, despite the very short drying times (typically around 1.0 to 1.5 s in the Büchi S-300 spray drier), a substantial fraction of alliin was converted to short-lived allicin and subsequently lost during particle formation.

### 3.4 Powder Stability Testing

Understanding the impact of relative humidity (RH) on the stability of powdered formulations is essential for their effective use in dry powder inhalers. It is particularly important to assess the long-term stability of encapsulated alliinase in 2F formulations under varying RH conditions. In the case of 3F formulations, where both alliin and the enzyme share the same carrier, elevated RH levels may trigger premature formation and loss of allicin, subsequently leading to a decrease in alliinase activity. Furthermore, the effect of increased RH on the fine particle fraction (FPF) was experimentally measured for both formulations, with and without the addition of leucine.

#### 3.4.1 Effect of Relative Humidity on the Specific Activity of Encapsulated Alliinase

The 2F particles (*W*_alliinase_ = 1%) were used to evaluate the enzyme’s response to storage across a wide range of relative humidities at room temperature. The results are presented in Figure 5A with the specific activity of stock lyophilized alliinase serving as a reference. Powders stored at 100% RH became moldy after 14 days and were not analyzed further.

**Figure 5:**
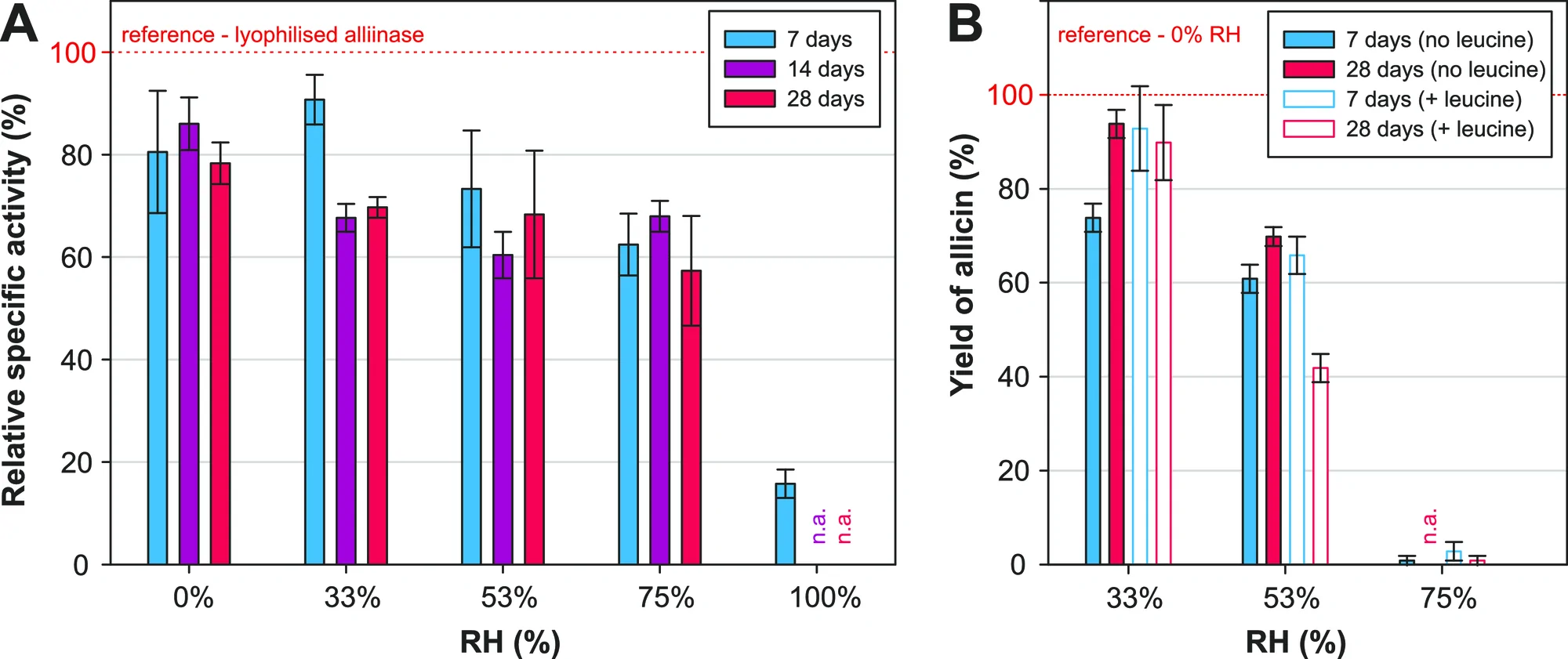
(A) Relative specific activity of encapsulated alliinase vs RH level and storage time (2F particles); (B) Effect of RH level and added leucine (10% *w/w*) on observed allicin yield (3F particles). Data are presented as mean ± SD, sample size (*n* = 3).

Under vacuum (0% RH), the relative specific activity of alliinase in the powders remained high, exceeding 80%, throughout the entire month. On the basis of our previous experience, encapsulated alliinase stored under these conditions remains highly active for more than three months. As anticipated, increasing the RH level and storage time led to a decrease in enzyme activity. Nevertheless, following a month of storage under conditions of high humidity (53% RH), the enzymatic activity remained above 60% of that observed for the lyophilized alliinase stock. These findings are very encouraging compared with our previously published data on the stability of free lyophilized alliinase,^22^ which indicated that the relative activity decreased to 50% after 7 days at 53% RH. This fact may be attributed primarily to the protective nature of amorphous maltodextrin used as a carrier material.

#### 3.4.2 Effect of Relative Humidity on the Powders Prepared Using the 3F Nozzle

The 3F particles (alliinase:alliin mass ratio 1:10, *W*_alliin_ = 1%, and feed_in:feed_out ratio 1:1 with alliin dissolved in the inner phase) were selected to evaluate allicin loss during storage at various RH levels. Additionally, the formulation with added leucine (10% *w/w*) was utilized to assess the effect of leucine on protecting the powder from excessive moisture uptake. The powder samples were analyzed for allicin content after complete dissolution using an identical formulation stored under vacuum as a reference.

Figure 5B indicates that as the RH level increases, the amount of allicin formed compared to reference particles stored at 0% RH decreases. Samples stored at 75% RH showed a significant loss of allicin content within the first week, suggesting that exposure to such high RH levels before use should be strictly avoided. Moreover, this also confirms that high air humidity is sufficient to trigger allicin formation. At such RH levels, 3F powders without leucine lost their structural integrity and could not be analyzed after 7 days. In contrast, powders with added leucine showed no visible signs of disintegration; however, most of the allicin was lost.

The presence of leucine was significantly beneficial after 7 days of storage, particularly at 33% RH, where it provided a short-term protective effect against the moisture uptake. However, after 28 days, the trend reversed: the leucine-free powders performed better in terms of the allicin yield than the leucine-added samples at 53% RH. This experiment suggests that during prolonged storage of 3F particles at RH higher than 33%, the presence of leucine in the formulation may not effectively prevent the spontaneous allicin formation.

#### 3.4.3 Effect of Relative Humidity on the Aerodynamic Properties of Prepared Powders

Storing powder in a humid environment may significantly affect not only the powder’s antibacterial performance, due to decreased allicin yield, but also its handling and aerodynamic properties, i.e., FPF. Therefore, the 3F formulations investigated in the previous section were stored for 4 weeks at 25 °C under vacuum or at 53% RH, and their FPF and moisture content were tested. This RH level was selected based on previous results indicating that it became limiting for allicin yield.

The initial moisture content of leucine-free 3F particles was 6.58 ± 0.04%. After 28 days at 0% RH and 53% RH, the equilibrium moisture content (EMC) was found to be 4.94 ± 0.06% and 10.42 ± 0.32%, respectively. For the particles with added leucine (10% *w/w*), the initial moisture content was 6.45 ± 0.33%, and the EMC at 0% RH and 53% RH was 4.47 ± 0.25% and 9.90 ± 0.13%, respectively. When compared, both 3F formulations showed very similar initial moisture content and EMC, regardless of leucine presence.

After 28 days of storage under vacuum, both formulations maintained their aerosolization properties, with the leucine-containing particles outperforming the basic formulation (Figure 6). However, the difference in FPF between the two formulations was halved compared with the initial value (day 0), while the performance of maltodextrin particles remained unchanged. Storage at 53% RH exhibited an even steeper decline in FPF maltodextrin particles with the addition of leucine. As a result, these powders outperformed the basic formulation.

**Figure 6:**
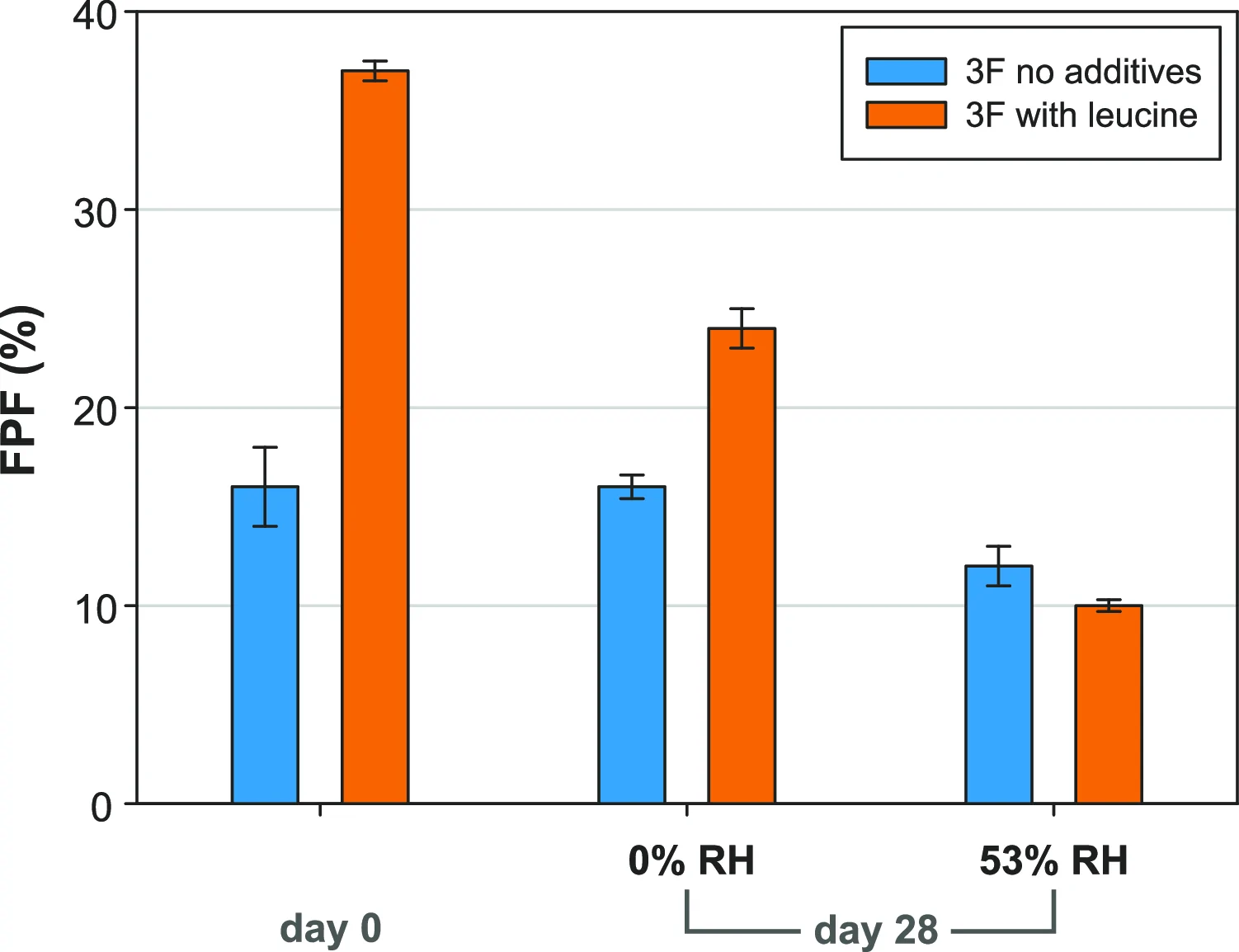
Fine particle fraction (FPF) of 3F powders containing either 0% or 10% *w/w* leucine, measured before and after 28 days of storage under 0% and 53% RH. Data are presented as mean ± SD, sample size (*n* = 3).

Overall, these results suggest that while leucine significantly enhances the initial and short-term aerodynamic properties, its effectiveness wanes when it is subjected to higher RH levels for extended periods. This diminished performance is likely due to an insufficient leucine content in the formulation, which results in moisture-induced structural changes such as the recrystallization of amorphous maltodextrin, thereby impairing powder aerosolization. It is important to note that, in practical applications, powders intended for DPIs are never exposed to such conditions as they are loaded into capsules and sealed in foil blisters under an inert atmosphere to protect them from elevated humidity, oxygen, and light.

#### 3.4.4 Antibacterial Activity of 3F Particles

The antimicrobial potential of allicin is primarily attributed to its thiol trapping capabilities and its engagement with a broad range of cellular targets. The electron-withdrawing effect of the oxygen atom creates electrophilic sulfur, which readily reacts with unrestricted thiol groups and Cys thiol residues, resulting in a depletion of the glutathione (GSH) pool (or bacillithiol pool found in *Bacillus* species), leading to the formation of S-allylmercaptoglutathione, the inactivation of important metabolic enzymes (S-thioallylation), and disruptions in cell-signaling pathways.^38,39^ The antibacterial effects of the 3F formulation, i.e., alliinase:alliin mass ratio 1:10, *W*_alliin_ = 1%, and feed_in:feed_out ratio 1:1 with alliin dissolved in the inner phase, were tested against *E*. *coli*. These formulations were tested both with and without added leucine (10% *w/w*), using the disk diffusion method and the contactless assay, allowing us to evaluate the activity of allicin vapors previously presented in our work.^19^

For the disk diffusion assay, 500 mg of powder was dissolved in 1 mL of PBS and left at room temperature for one hour to achieve complete alliin conversion. The 250 mg·mL^–1^ and 125 mg·mL^–1^ particle concentrations were achieved by dilution. Finally, 20 μL of solution was applied to a paper disk placed on the agar plates. Considering complete alliin conversion, the theoretical allicin amounts applied per disc ranged from 11.4 to 45.7 μg. As expected, the inhibition zone diameter was concentration-dependent, increasing with allicin content (Figure 7A and Figure 7B). Moreover, no significant difference was observed between the two formulations (Tab. SI 1), indicating that once the powder is fully dissolved, the presence of leucine has no adverse effect on allicin production in the fully dissolved state.

**Figure 7:**
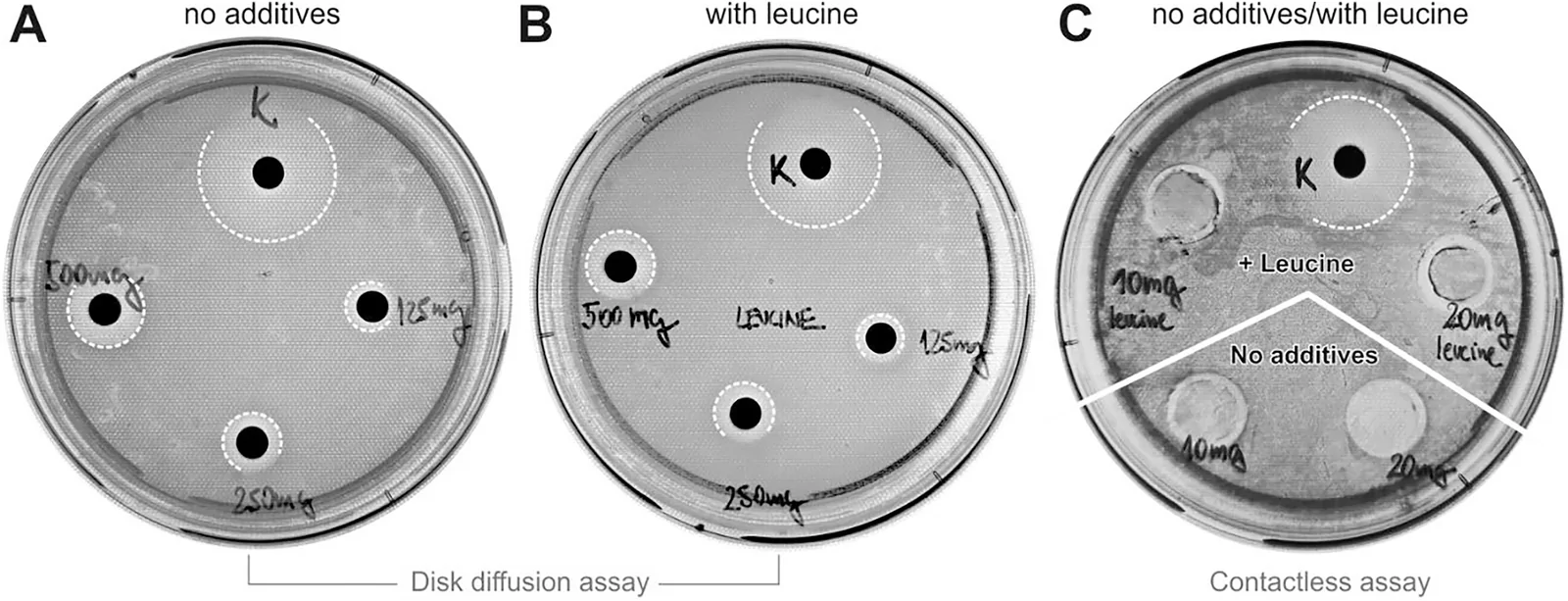
Disk diffusion tests showing the antibacterial activity of dissolved 3F formulations, without (A) and with leucine (B), and contactless assay (C) with the same formulations in a dry state against *E. coli*. Note: Kanamycin solution (K) was applied to the paper disk in all cases as a positive control. All experiments were done in triplicate.

In the contactless assay (Figure 7C), 10 and 20 mg of dry 3F powder were placed into a 3D-printed holder to evaluate the antibacterial activity of allicin vapors, which were generated as a result of absorption of air moisture by the particles placed above the agar layer. Figure 7C illustrates a clear distinction between the two formulations: powders containing added leucine (top section) showed no antibacterial effect after 24 h of incubation, meaning that no inhibition zone formed beneath the holder (only the imprint of the holder is visible). This observation underscores the short-term moisture protective properties of hydrophobic leucine. In contrast, the formulation without leucine demonstrated partial (10 mg) and complete (20 mg) inhibition of the bacterial growth. However, for deep lung delivery, the presence of leucine should not adversely affect allicin formation, as the mucus-lined surface of the respiratory tract creates an environment that favors the rapid dissolution of deposited particles.

### 3.5 Effect of Allicin on Cell Viability

Figure 8 summarizes the cytotoxic effect of allicin on the A549 cell line following exposure to spray-dried powders produced by the 2F or 3F nozzle. Cell viability was assessed after two exposure durations (1 and 4 h), followed by a total incubation time of 24 h. The estimated total allicin concentration in each sample ranged from 0.78 to 100 μg·mL^–1^, based on the assumption of 100% enzymatic conversion of alliin.

**Figure 8:**
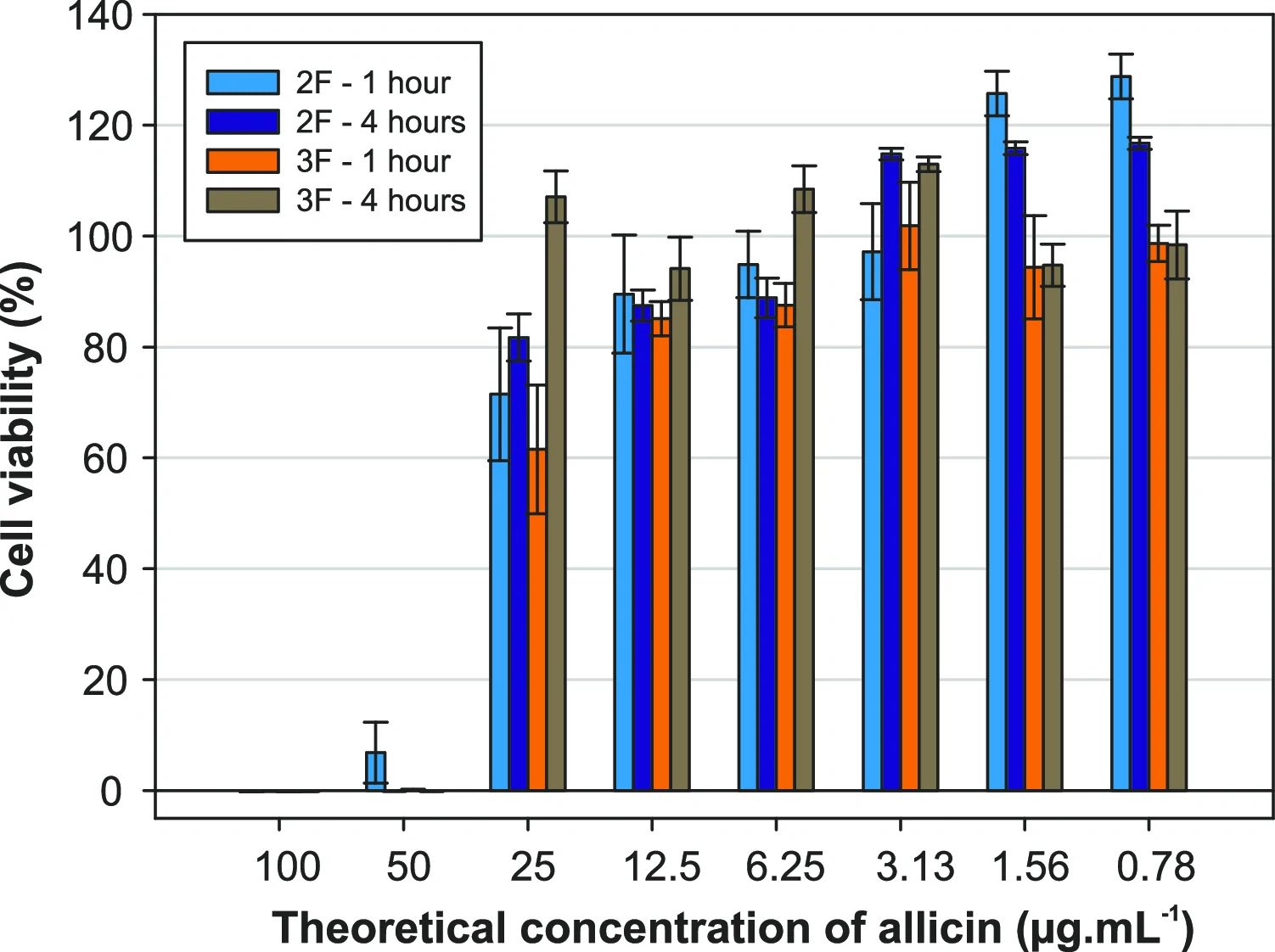
Viability of A549 cells after exposure to binary 2F mixture and 3F particles. The *x*-axis shows the estimated allicin concentration, calculated assuming complete alliin conversion. The *y*-axis represents cell viability, expressed as a percentage of the untreated control. Data are presented as mean ± SD, sample size (*n* = 6).

A concentration-dependent reduction in cell viability was observed for both formulations. At the highest allicin concentration (100 μg·mL^–1^), all samples induced near-complete cytotoxicity, with cell viability falling below 10% under all conditions. When the allicin amount was reduced to 50 μg·mL^–1^, severe cytotoxic effects persisted in most conditions, with three of the four samples showing viability close to 0%. The only exception was the 3F sample, which showed a modest increase in viability after 4 h of exposure, but overall cytotoxicity remained high. At 25 μg·mL^–1^, viability improved substantially in all cases, generally exceeding 60%. In the lower concentration range (12.5 to 0.78 μg·mL^–1^), most samples maintained high viability (80 to 120%). The lowest concentrations tested (1.56 and 0.78 μg·mL^–1^) resulted in viability values near or above 100%, indicating minimal or no cytotoxicity at these concentrations.

To quantify the cytotoxic potential of *in situ*-formed allicin, IC_50_ values were calculated for each formulation and exposure duration. For the 2F nozzle formulation (physical mixture of alliin and alliinase particles), the IC_50_ was 28.8 μg·mL^–1^ following 1 h of exposure and 29.2 μg·mL^–1^ after 4 h. For the 3F nozzle formulation, the IC_50_ was 27.9 μg·mL^–1^ after 1 h and increased to 38.2 μg·mL^–1^ after 4 h. The time-dependent reduction in effective cytotoxicity for the 3F formulation may be due to a higher rate of allicin degradation or to cellular recovery processes.

The results of this study indicate that high concentrations of *in situ*-formed allicin significantly reduce the viability of A549 lung epithelial cells. These findings are consistent with the previously reported cytotoxic effects of allicin and allicin-releasing compounds. For example, Kharazmi et al. described a concentration-dependent suppression of cell proliferation associated with G2/M cell cycle arrest,^40^ supporting the current observations of strong cytotoxic effects at higher allicin concentrations.

In this study, cytotoxicity was primarily dependent on the allicin concentration, while exposure time had only a limited influence. This scenario was expected, given allicin’s short half-life due to its high reactivity and instability. In our preliminary experiments, we also tested a 24-h exposure but observed no substantial difference in cell viability compared to the 1-h and 4-h time points. The relatively stable IC_50_ values observed between these time points suggest that a short initial exposure to allicin is sufficient to trigger a cytotoxic response and that prolonged incubation does not enhance the effect, possibly due to partial degradation of allicin over time and rapid initiation of cellular damage.

The IC_50_ values determined in this study ranged from 27.9 μg·mL^–1^ (172 μM) to 38.2 μg·mL^–1^ (236 μM), defining the cytotoxic threshold of allicin for A549 cells under the given experimental conditions. These results provide an important reference for formulation development and dose selection in future pulmonary delivery studies. For comparison, the reported LD_50_ values for *E. coli* and *S. aureus* are 15 μg·mL^–1^ and 12 μg·mL^–1^, respectively.^41^

It is important to note that allicin is rapidly inactivated in the human body by endogenous thiol-containing antioxidants such as glutathione (GSH),^42^ which is present in very high concentrations in the epithelial lining fluid of the lungs as protection against oxidative stress (approximately 100 times higher GSH levels than in blood).^43,44^ We therefore assume that allicin-induced cytotoxicity would be substantially reduced *in vivo* because of natural detoxification mechanisms. This hypothesis remains to be tested in follow-up coculture *in vitro* models supplemented with physiologically relevant concentrations of glutathione, which falls outside the scope of the current study but will be addressed in our future work.

## 4 Conclusions and Prospects

Allicin is widely acknowledged as one of the most powerful natural substances; however, its high reactivity and low stability restrict its practical use. By facilitating the production of allicin from its stable precursors *in situ*, we can overcome most delivery and stability challenges, allowing us to fully utilize its therapeutic potential for pulmonary use.

In our approach, we demonstrate that a three-fluid nozzle (3F) can solve these problems by ensuring that most particles contain both the enzyme and the substrate needed for the independent *in situ* production of allicin upon deposition. For the 3F nozzle, it was found that among all examined parameters, the spatial distribution of the substrate and enzyme during atomization played a crucial role in the unwanted allicin production during the spray-drying process. Specifically, switching alliinase from the outer to the inner phase resulted in a more than 20% decrease in the observed allicin yield.

Upon dissolution, the 3F particles—both with and without added leucine—showed similar antibacterial effects in disk diffusion assays. In contrast, noncontact assays assessing the antibacterial activity of allicin vapors indicated that the presence of hydrophobic leucine inhibits spontaneous allicin production triggered by moisture absorption from the air for 24 h. This is unlikely to pose an issue for pulmonary delivery, as the omnipresent airway surface liquid ensures rapid particle dissolution and allicin formation. Due to its volatility, locally produced allicin may also reach areas typically inaccessible to inhaled particles, as is often the case in patients with small airway dysfunction. This hypothesis will be validated in upcoming studies that simulate the relevant physiological microenvironment of the respiratory tract. Additionally, coencapsulation of stabilized precursors may serve as a promising delivery strategy for other unstable substances that have been deemed unfit for practical use.

## Supplementary Material



## Data Availability

All data are available from the corresponding author upon reasonable request.

## Notes

Additional experimental results for 2F and 3F pure D-mannitol, and maltodextrin particles with or without additives (ammonium carbonate, leucine) are presented in Figures SI 1 to SI 11. Results of disk diffusion assays for selected 3F formulations are summarized in Table SI 1 (PDF)
