# Supplementary material for: Inhalable Spray-Dried Carriers for Deep Lung Delivery and In Situ Synthesis of Allicin
Source: ACS Omega. 2026 Aug 5;11(32):48144–56. doi: 10.1021/acsomega.6c04308 (PMC13491919; doi:10.1021/acsomega.6c04308)
Supplement: Supplementary file 1 [file ao6c04308_si_001.pdf]

Supporting Information

Inhalable spray-dried carriers for deep lung delivery and in situ synthesis of allicin

*Lucie Mašková, Mária Kalina, Karolína Slonková, Lara Mugrauer, Vojtěch Klimša, Anna Reiserová, Kateřina Slaninová, Denisa Lizoňová, Viola Tokárová, Ondřej Kašpar**

Department of Chemical Engineering, University of Chemistry and Technology Prague, Technická 5, Prague 6, 166 28, Czech Republic

**Corresponding author: kasparo@vscht.cz*

2F and 3F particles fractionated by 8-stage ACI at a flow rate of 60 L.min^{-1}

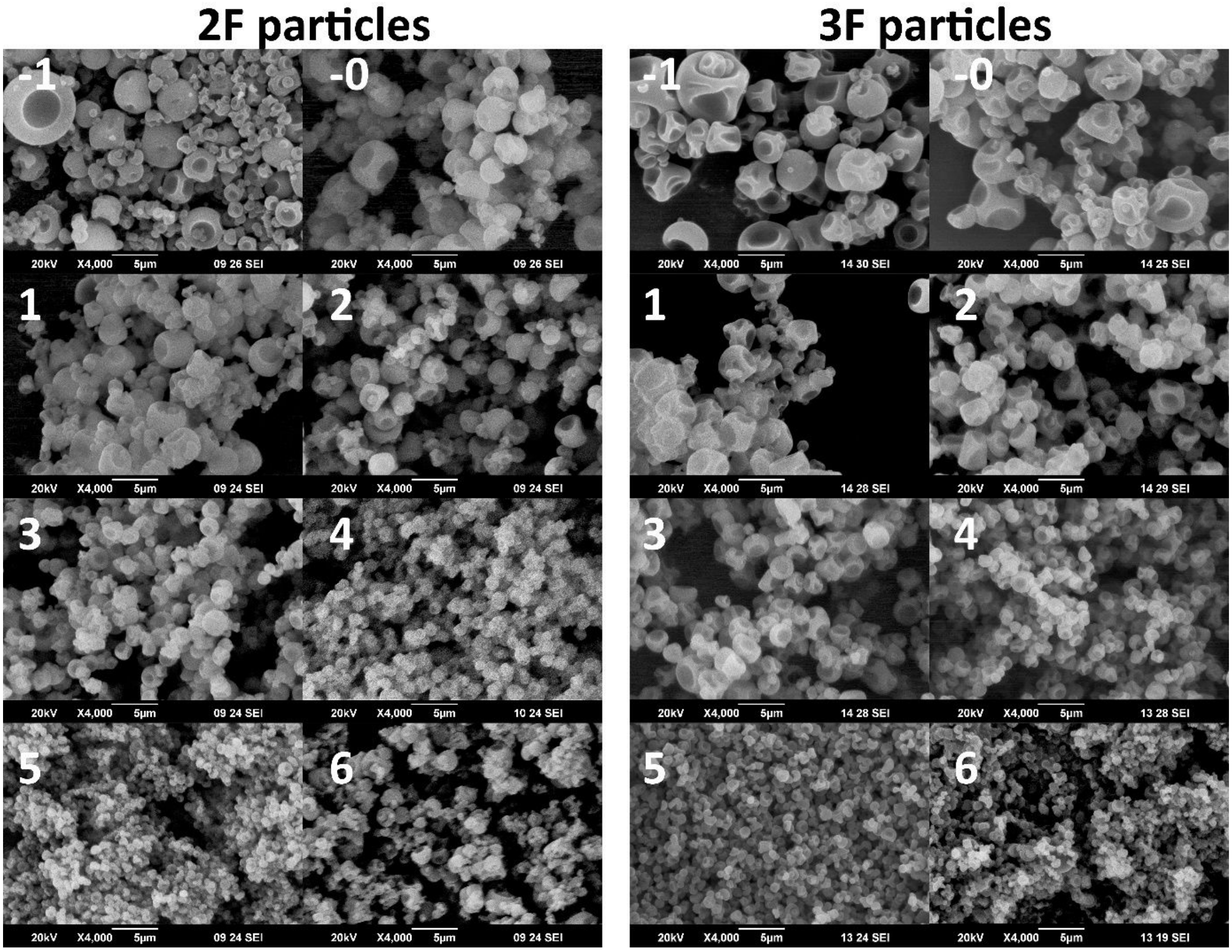

Fig. SI 1 SEM images of 2F (sample 1) and 3F (sample 3) maltodextrin particles deposited on stage no. -1 to 6 of ACI at a flow rate of 60 L.min^{-1}.

FPF vs nozzle and carrier type

Fig. SI2 shows the improvement in FPF after the addition of 10% *w/w* leucine or 30% *w/w* ammonium carbonate, compared to pure maltodextrin and D-mannitol (for illustration). Leucine performed notably better than ammonium carbonate, with the enhancement being even more significant for the 3F nozzle, leading to a 2.6-fold increase in FPF (**Fig. SI 7** and **Fig. SI 8**).

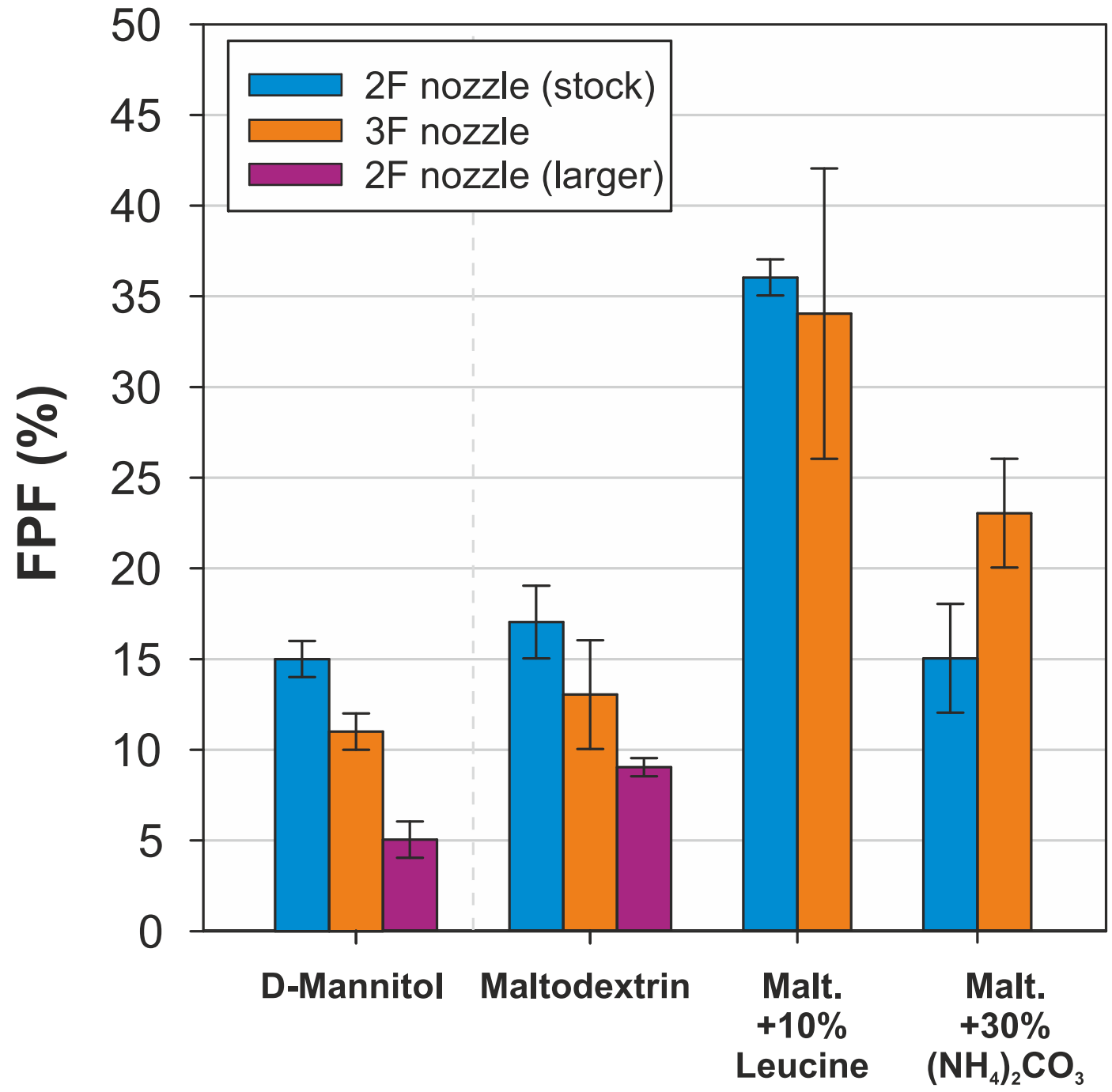

Fig. SI 2 FPF for D-mannitol and maltodextrin (without and with additives) spray-dried particles prepared using 2F (stock and larger) and 3F nozzles. Data are presented as mean ± SD, sample size (n = 3).

3F particles with added ammonium carbonate

Surface morphology and PSD

The particle size distribution (PSD) curves and SEM images displaying the particle morphology are shown in **Fig. SI 3** and **Fig. SI 4**, respectively.

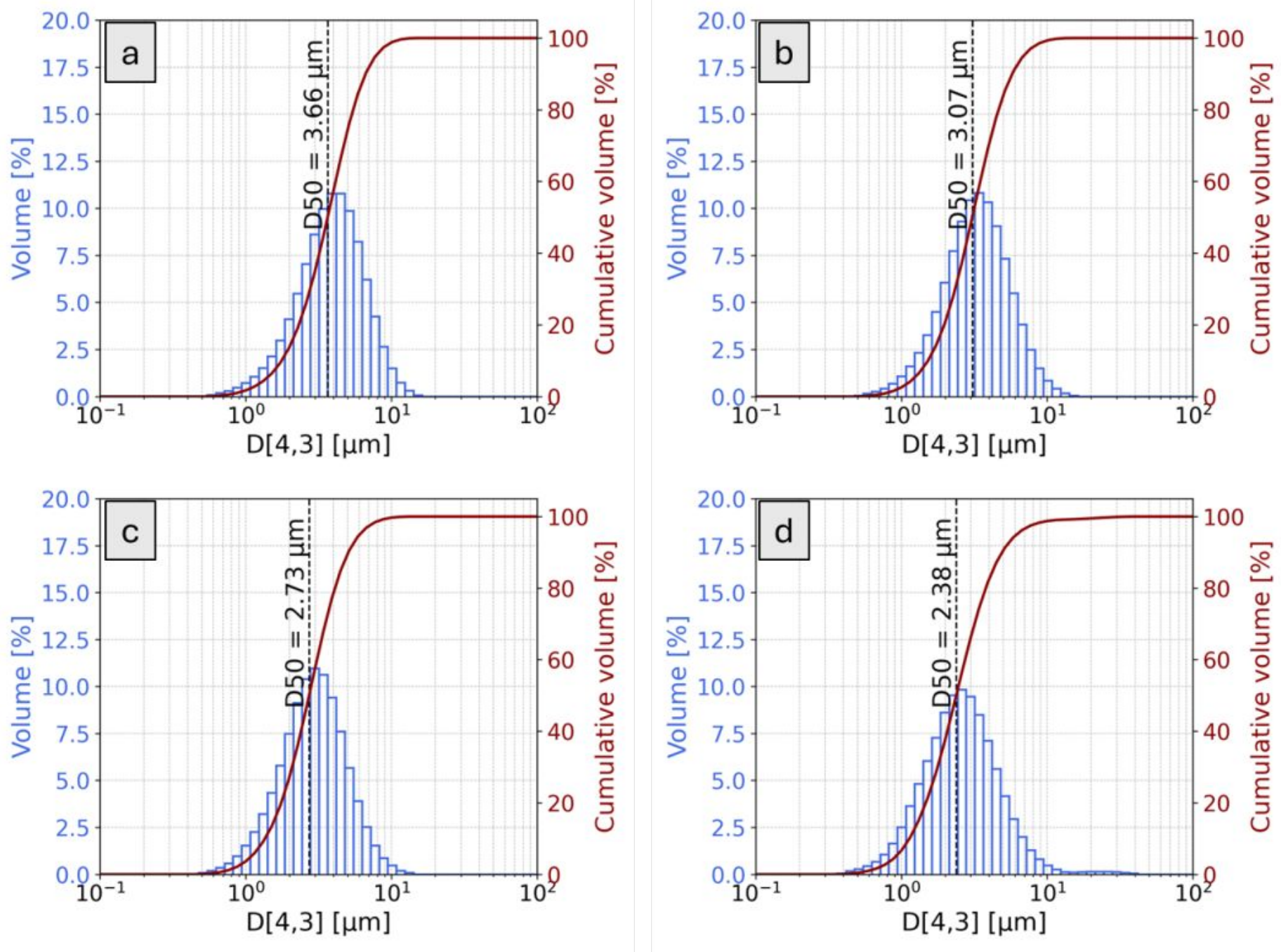

Fig. SI 3 PSD of spray-dried samples with added ammonium carbonate: (a) 0% *w/w*, (b) 1% *w/w*, (c) 10% *w/w*, and (d) 30% *w/w*.

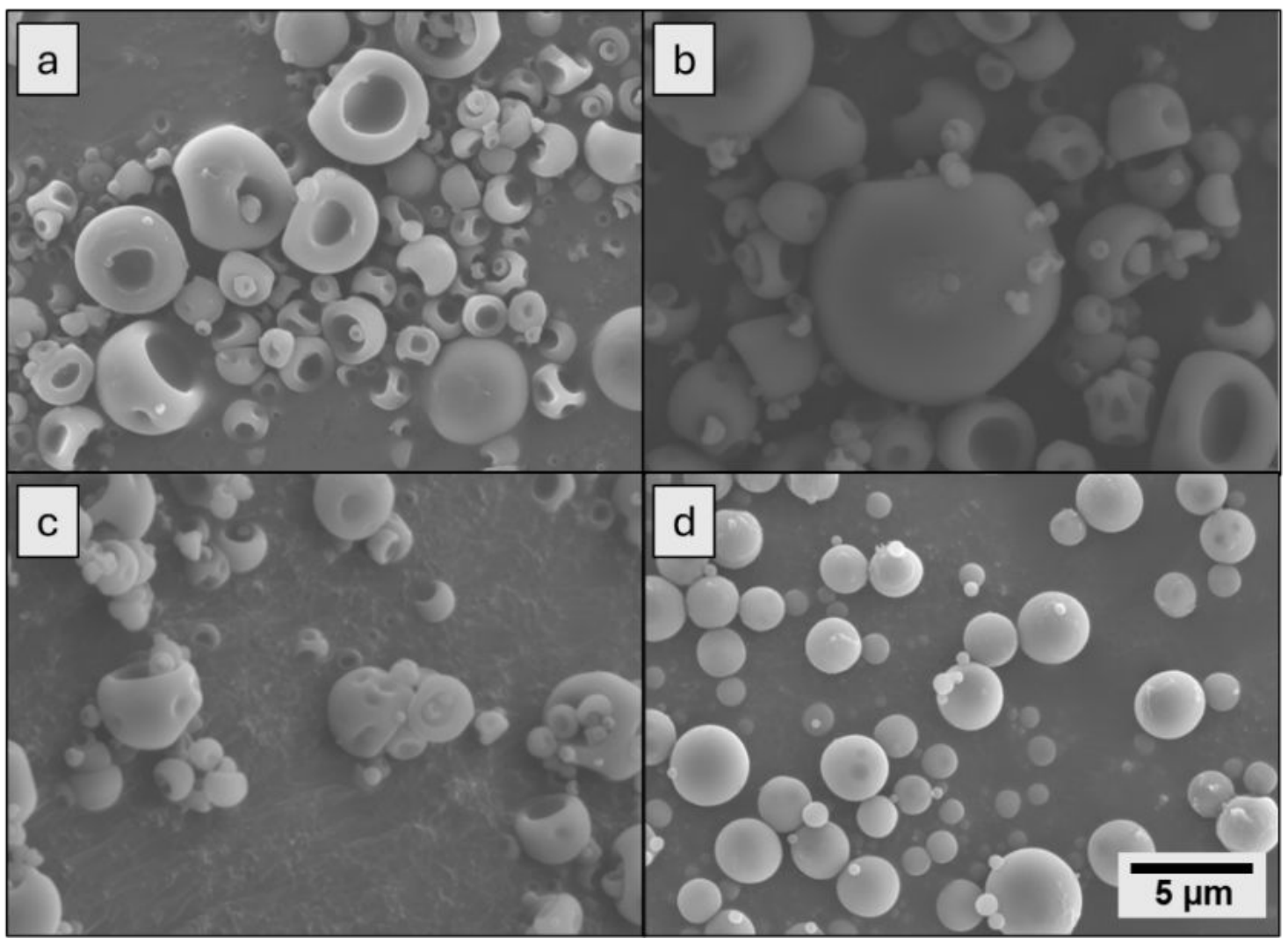

Fig. SI 4 SEM images of 3F particles with added ammonium carbonate: (a) 0 % *w/w*, (b) 1% *w/w*, (c) 10% *w/w*, and (d) 30% *w/w*.

As porogen concentration increases, the average particle size decreases slightly, and the particle shape transitions from buckled surfaces to smooth spheres. This change in morphology can be attributed to the higher porosity of the particle walls, which prevents pressure buildup and collapse during the drying process. At low to moderate porogen levels (up to 10% *w/w*), the PSD remains unaffected, whereas at higher concentrations (30% *w/w*), the presence of porogen results in broader PSDs and the formation of agglomerates.

3F particles with added leucine

Surface morphology and PSD

The particle size distribution curves and SEM images displaying the particle morphology are shown in **Fig. SI 5** and **Fig. SI 6** (respectively). Leucine acts as a surface-active excipient, preferentially occupying the droplet-air interface and forming a protective hydrophobic crystalline shell upon drying. While its presence can aid dispersibility, an excess of leucine leads to surface rigidity and internal collapse, particularly at concentrations of 5% *w/w* and 15% *w/w*, as observed in the SEM images. An intermediate concentration (10% *w/w*) was chosen as the optimal balance between structure and uniformity.

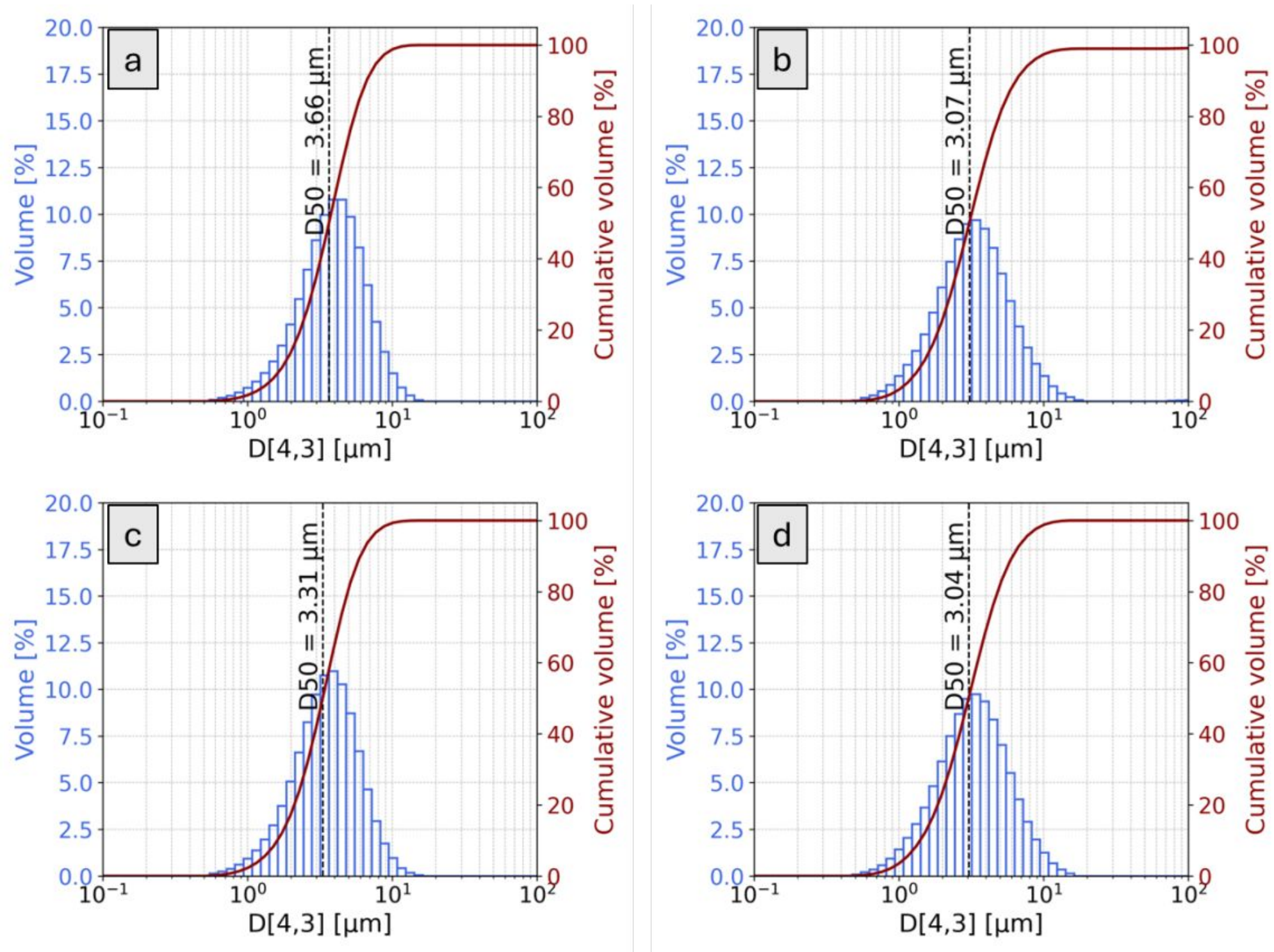

Fig. SI 5 PSD of 3F particles with the addition of leucine: (a) 0% *w/w*, (b) 5% *w/w*, (c) 10% *w/w*, and (d) 15% *w/w*.

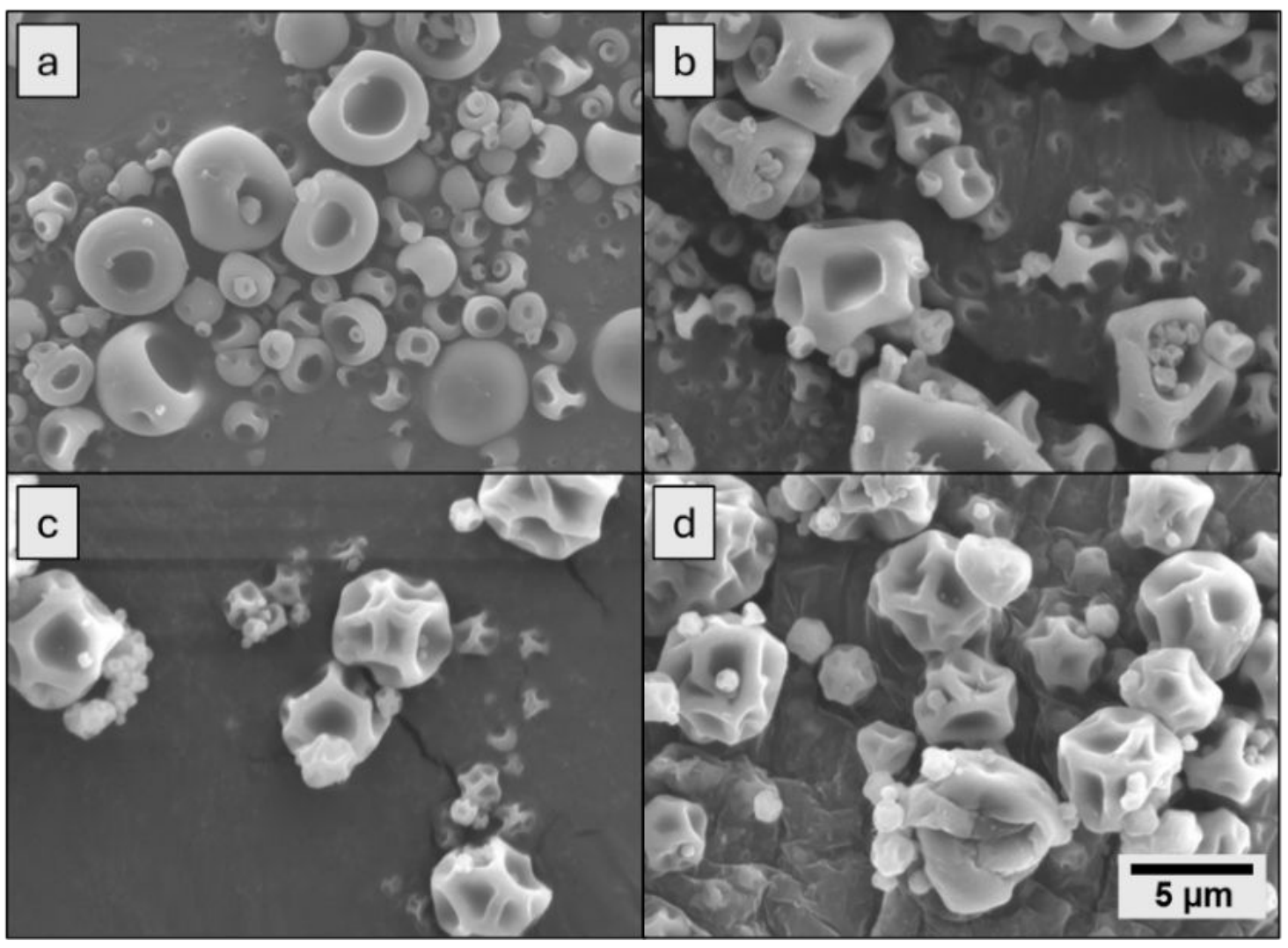

Fig. SI 6 SEM images of 3F particles with the addition of leucine: (a) 0% *w/w*, (b) 5% *w/w*, (c) 10% *w/w*, and (d) 15% *w/w*.

Aerosolisation properties of 3F particles

Aerosolisation properties vs ammonium carbonate content

The results in **Fig. SI 7** indicate that increasing the ammonium carbonate amount steadily improves the fine particle fraction (FPF), rising from 13% at 0% *w/w* to 23% at 30% *w/w*. This trend suggests that ammonium carbonate enhances particle aerosolisation, likely by increasing porosity and decreasing particle cohesion. However, its pungent odour and tendency for powders to clump during handling (often resulting in reduced emitted doses) make its use undesirable in our specific case, despite the improved aerosolisation.

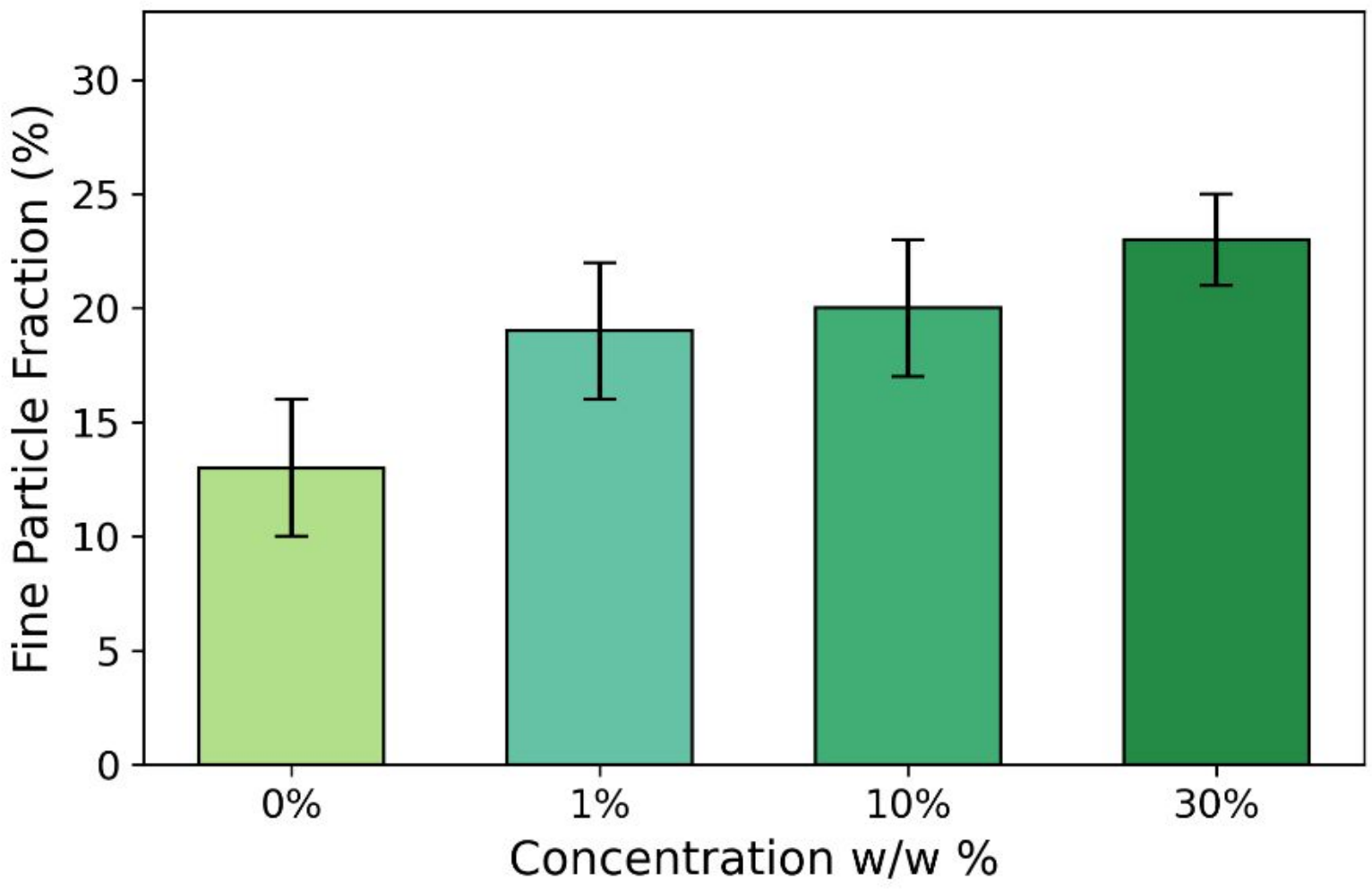

Fig. SI 7 Effect of ammonium carbonate concentration on fine particle fraction (FPF) of 3F particles.

Aerosolisation properties vs leucine content

The results from ACI measurements for 3F particles with increasing content of leucine are shown in the **Fig. SI 8.**

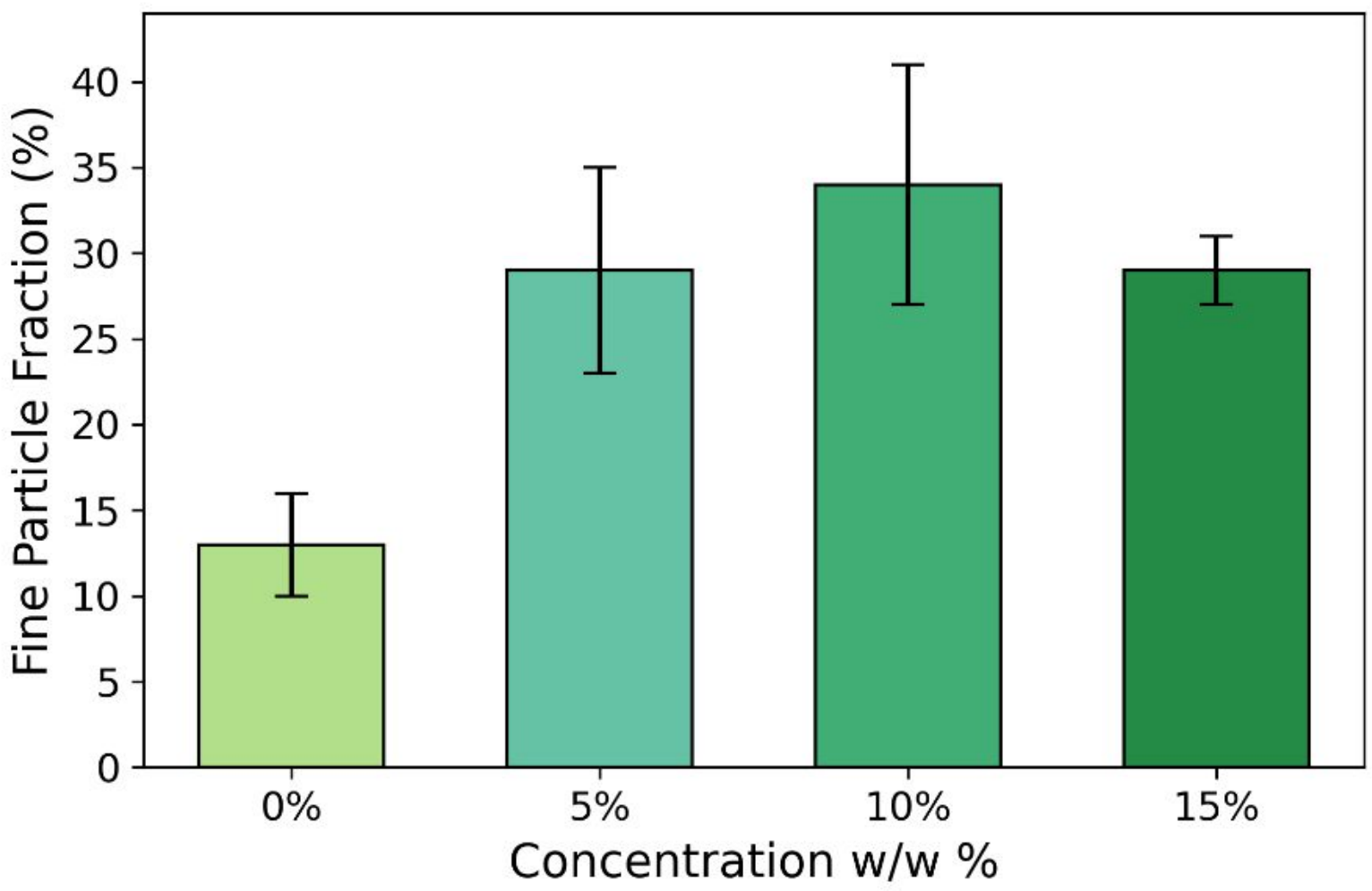

Fig. SI 8 Effect of leucine concentration on fine particle fraction (FPF) of 3F particles.

Spatial distribution of inner and outer phases

Fluorescent dyes were used as visual tracers to assess the spatial distribution of the two phases and verify whether core-shell particles are produced using the 3F nozzle. Rhodamine B and fluorescein sodium salt were added to the outer and inner feeds, respectively. The dye localisation was observed using fluorescence confocal microscopy (**Fig. SI 9**). Despite the limitation of the used optics, the composite image reveals that the majority of particles contain both dyes.

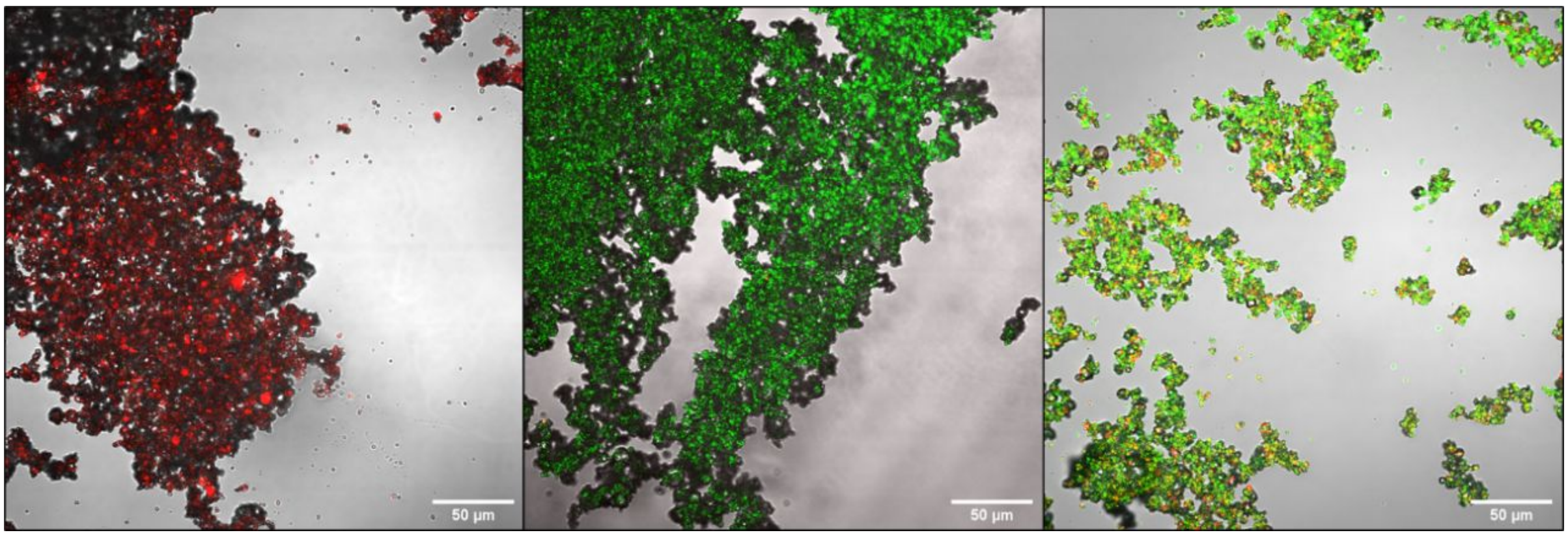

Fig. SI 9 Fluorescence confocal microscopy images of 3F particles, with rhodamine B (red) added to the outer phase and fluorescein (green) added to the inner phase. The left panel shows the red fluorescence channel (rhodamine). The middle panel displays the green fluorescence channel (fluorescein), corresponding to the inner phase. The right panel is the overlayed image.

Flow rate ratio vs 3F particle morphology

Since the feed ratio can influence droplet formation and thus powder characteristics, both surface morphology (**Fig. SI 10**) and PSDs (**Fig. SI 11**) of 3F powders were examined.

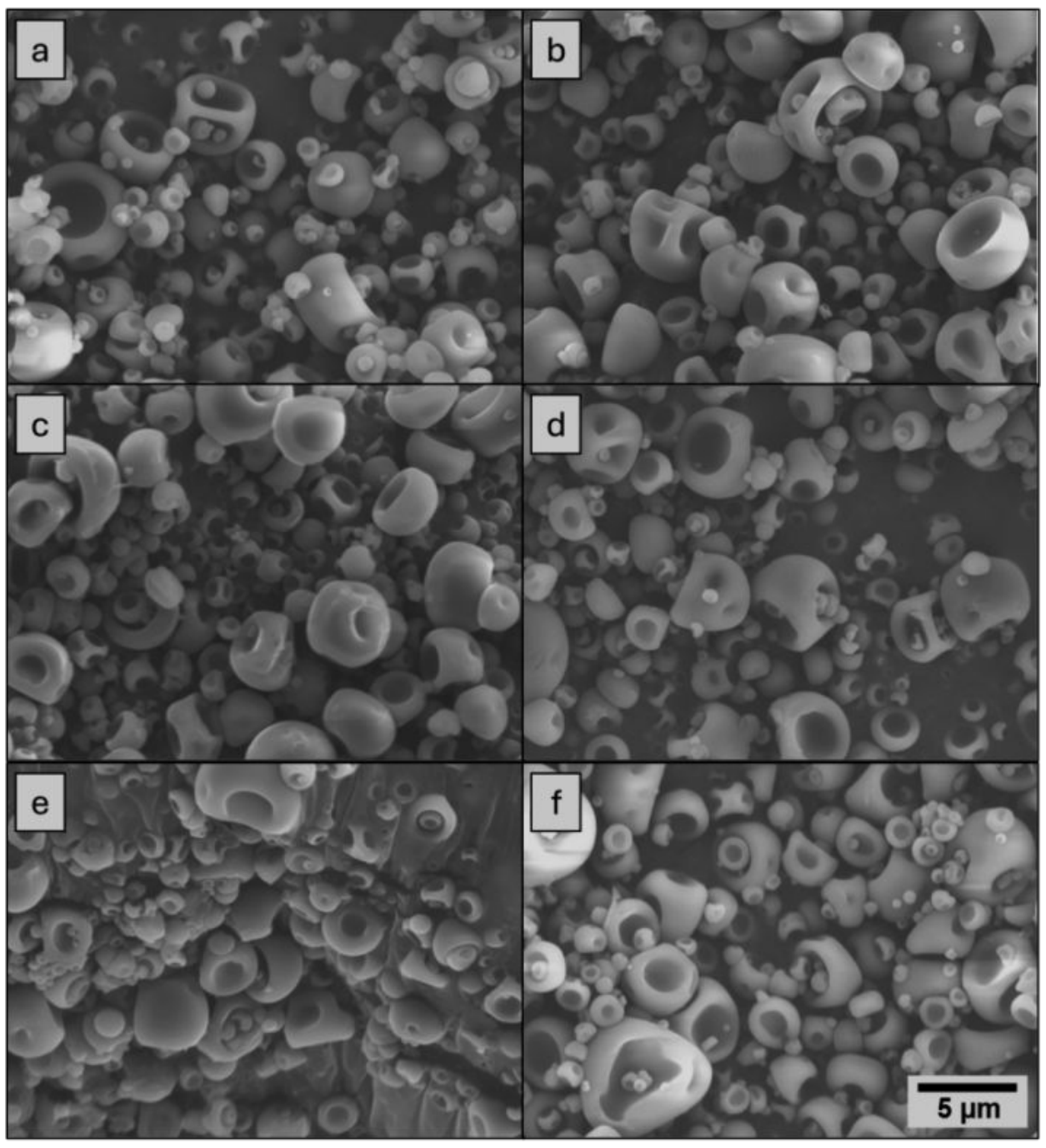

Fig. SI 10 SEM images of spray-dried particles produced with varying inner:outer phase flow rate ratios using 3F nozzle: (a) 1:9, (b) 1:2.3, (c) 1:1, (d) 2.3:1, (e) 9:1, and (f) 1:1 (phases reversed).

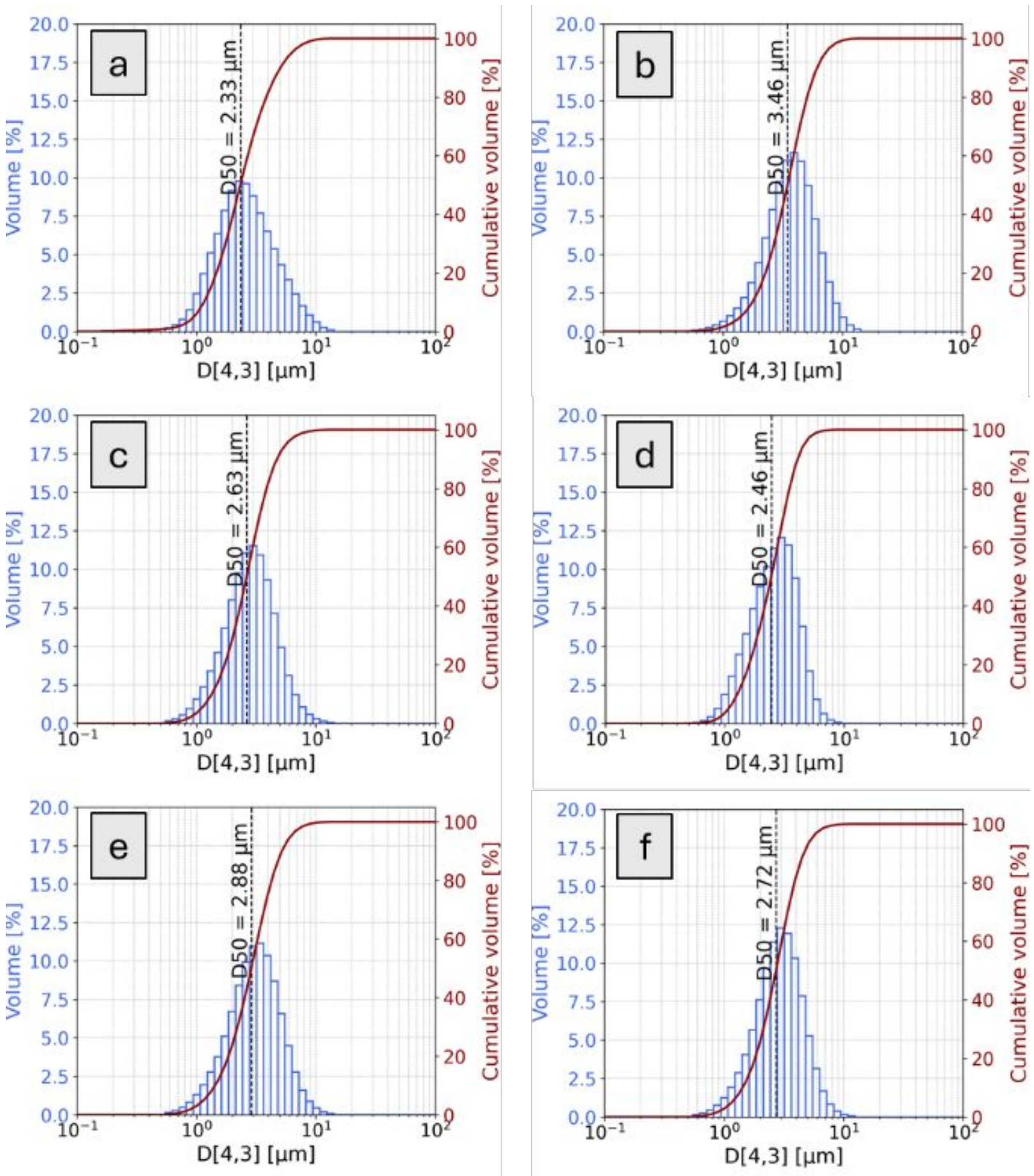

Fig. SI 11 PSD of 3F samples prepared with different inner:outer phase flow rate ratios: (a) 1:9, (b) 1:2.3, (c) 1:1, (d) 2.3:1, (e) 9:1, and (f) 1:1 (phases reversed).

Disk diffusion tests

Tab. SI 1 Comparison of antibacterial activity of dissolved 3F formulations

Samples concentration [mg.mL^{-1}]	Relative inhibition zone diameter d/d_kanamycin [%]	
	Without leucine	With leucine
500	51.2 ± 4.5	51.5 ± 1.4
250	41.4 ± 4.0	44.5 ± 1.2
125	31.2 ± 0.4	36.4 ± 1.2
